# ‘*Candidatus* Liberibacter asiaticus’ Multimeric LotP Mediates *Citrus sinensis* Defense Response Activation

**DOI:** 10.3389/fmicb.2021.661547

**Published:** 2021-08-04

**Authors:** Marcelo L. Merli, Kaylie A. Padgett-Pagliai, Alexandra E. Cuaycal, Lucila Garcia, Maria Rosa Marano, Graciela L. Lorca, Claudio F. Gonzalez

**Affiliations:** ^1^Department of Microbiology and Cell Science, Genetics Institute, Institute of Food and Agricultural Science, University of Florida, Gainesville, FL, United States; ^2^Instituto de Biología Molecular y Celular de Rosario (IBR), Consejo Nacional de Investigaciones Científicas y Tecnológicas, Rosario, Argentina

**Keywords:** Huanglongbing (HLB), citrus greening disease, effector protein, proteomics, pathogen

## Abstract

‘*Candidatus* Liberibacter asiaticus’ is known as the most pathogenic organism associated with citrus greening disease. Since its publicized emergence in Florida in 2005, ‘*Ca*. L. asiaticus’ remains unculturable. Currently, a limited number of potential disease effectors have been identified through *in silico* analysis. Therefore, these potential effectors remain poorly characterized and do not fully explain the complexity of symptoms observed in citrus trees infected with ‘*Ca*. L. asiaticus.’ LotP has been identified as a potential effector and have been partially characterized. This protein retains structural homology to the substrate binding domain of the Lon protease. LotP interacts with chaperones like GroEL, Hsp40, DnaJ, and ClpX and may exercise its biological role through interactions with different proteins involved in proteostasis networks. Here, we evaluate the interactome of LotP—revealing a new protein–protein interaction target (Lon-serine protease) and its effect on citrus plant tissue integrity. We found that via protein–protein interactions, LotP can enhance Lon protease activity, increasing the degradation rate of its specific targets. Infiltration of purified LotP strained citrus plant tissue causing photoinhibition and chlorosis after several days. Proteomics analysis of LotP tissues recovering after the infiltration revealed a large abundance of plant proteins associated with the stabilization and processing of mRNA transcripts, a subset of important transcription factors; and pathways associated with innate plant defense were highly expressed. Furthermore, interactions and substrate binding module of LotP suggest potential interactions with plant proteins, most likely proteases.

## Introduction

One of the causal agents of Huanglongbing (HLB or citrus greening disease) is ‘*Candidatus* Liberibacter asiaticus.’ This bacterium is known to be transmitted by psyllids and resides within the phloem of the citrus tree where it causes the incurable plant disease. The characteristic symptoms of HLB in the citrus host include blotchy and mottled leaves, small and deformed fruits, and yellow shoots. Consequently, infected trees suffer shorter life span and premature fruit drop associated with a reduction in marketable quality (Bové, 2006). The disease has already devastated the citrus industry worldwide. In Florida alone, one of foremost orange juice producers of the world, the citrus production value for the 2018–2019 season was USD 873 million, 37% higher than the 2017–2018 crop (Fried and Hudson, 2020). However, the 2017–2018 season was USD 551 million, 41% less than the 2016–2017 season and the lowest since 1976–1977 (USDA, 2018). As Florida’s top agricultural product, fruit yield inconsistency due to HLB has caused catastrophic downsizing to the entire industry. From 2002 to 2017, the number of citrus growers decreased from 7,389 to 2,775 (Veneman et al., 2004; Perdue and Hamer, 2017), while the number of juice processing facilities decreased from 41 in 2003/2004 to 14 in 2016/2017 (Singerman et al., 2018).

‘*Ca.* L. asiaticus’ remains unculturable, and the efforts to maintain the bacterium in axenic cultures have been unsuccessful. Its closest phylogenetically culturable microorganism is *Liberibacter crescens* (Leonard et al., 2012) and has been widely used as a surrogate strain to conduct basic research. However, *L. crescens* is a free-living bacterium whose genome encodes ∼250 additional genes when compared against ‘*Ca.* L. asiaticus’ (Fagen et al., 2014). Therefore, the limited scientific understanding of ‘*Ca.* L. asiaticus’ physiology relies on theories grounded on interpretation and analysis of transcriptome and proteomic data. Unfortunately, many genes present in the ‘*Ca.* L. asiaticus’ genome encode for hypothetical proteins, which thwart a rational analysis regarding important physiological pathways. Nevertheless, a limited number potential disease effectors have been identified: a group of ATP translocases enables the bacterium to act as an “energy parasite” (Vahling et al., 2010); a prophage-encoded peroxidase, SC2, that eliminates H_2_O_2_ synthesized by the plant as a defensive mechanism (Jain et al., 2015); and CLIBASIA_05315, which was shown to co-localize with chloroplasts and with feeble association with callose deposition (Pitino et al., 2016). This protein was later associated with the capacity to inhibit proteases by physical interaction and renamed SDE1 (Clark et al., 2018). In addition, two secreted proteins, CLIBASIA_00460 and short synthesized candidate peptides, were able to enhance reactive oxygen species biosynthesis and chlorosis in citrus plants (Liu et al., 2019; Chen et al., 2020).

Despite efforts, none of the aforementioned proteins can fully explain the complexity of symptoms observed in infected citrus trees. Still, mechanisms underlining the pathogenicity process remain largely unknown. A complete understanding of the etiology of this disease will depend on the identification and full characterization of all pathogenic determinants. The proteolysis network and how it is regulated are critical to explain many aspects of the disease (Franco et al., 2020). Elucidating the role of proteins expressed during initial citrus infection, as the bacteria is injected into the phloem, will contribute to our fundamental knowledge of this disease, potentially aiding in the design of future strategies to control HLB.

Recently, a newly described protein has been identified and partially characterized as a potential effector in HLB (Loto et al., 2017). *CLIBASIA_03135* (*lotP*) belongs to the LdtR regulon (Pagliai et al., 2017) and was identified by transcriptomic analysis as one of the highest induced genes in ‘*Ca.* L. asiaticus’ (seven-fold induction) during the invasion of the citrus plants (Yan et al., 2013). Given the steep upregulation of *lotP* when ‘*Ca.* L. asiaticus’ infects the citrus host, we have published primary evidence supporting the role of this protein in the bacterium’s adaptation response to environmental stress. The encoded protein was partially characterized and renamed as LotP (Loto et al., 2017). Surprisingly, this protein, while retaining structural homology to the singular substrate binding domain of the Lon protease, showed no chaperone activity. However, we demonstrated that LotP interacts with central *Escherichia coli* and *L. crescens* chaperones like GroEL, Hsp40, DnaJ, and ClpX. These characteristics suggest that LotP may exercise its biological role through interactions with other proteins involved in proteostasis networks. Still, the mechanism involved in protein–protein interaction remains to be elucidated.

The aim of this work was to evaluate the importance of critical amino acids affecting LotP folding and to assess the importance of the structural changes mediating LotP interaction with other proteins. Globular N-terminal domain of LotP is critical in the formation of stable dimers and active multimers. We used the structural traits discovered to identify a new member of the LotP interactome. The evidence collected demonstrates that LotP can interact with the ‘*Ca.* L. asiaticus’ Lon protease, boosting its catalytic activity *in vivo*. Because of the critical contribution of serine proteases to the etiology of the citrus greening disease, we have evaluated the potential effects of LotP directly on plants. To this end, we conducted pure protein infiltration assays using purified protein into healthy citrus leaves. We have demonstrated that LotP induces leaves chlorosis associated with a transient negative impact on photosynthesis. Proteomic analysis of the infiltrated leaves revealed a significant plant reaction with clear effects on proteins responding to pathogenic processes.

## Materials and Methods

### *In silico* Analysis

The protein-conserved residues were determined from alignments using PSI-BLAST^1^. The multiple sequence alignment and figure was generated with MEGA6 (Tamura et al., 2013) and BOXSHADE version 3.21^2^. The LotP dimer structure was modeled using GalaxyWEB sever (Ko et al., 2012; Shin et al., 2014).

### Strains and Media

The strains used in this work are listed in Table 1. *E. coli* strains were cultured at 37°C in Lysogeny broth (LB) medium or M9 media supplemented with 0.1 mM of CaCl_2_, 2 mM of MgCl_2_, and 0.2% v/v glycerol. The culture media were amended with the appropriate concentration of antibiotics as recommended for each genetic vector used (Table 1).

**TABLE 1:** Strains and plasmids used in this study.

Strains	Relevant genotype	References
*Escherichia coli* DH5α	F^–^ φ80*lac*ZΔM15 Δ(*lac*ZYA-*arg*F)U169 *rec*A1 *end*A1 *hsd*R17(r_*K*_^–^, m_*K*_^+^) *pho*A *sup*E44 λ^–^ *thi*-1 *gyr*A96 *rel*A1	Laboratory stock
*E. coli* BL21 (DE3)	F^–^ *omp*T *gal dcm lon hsd*SB(r_*B*_- m_*B*_-) λ(DE3 [*lac*I *lac*UV5-T7 *gene* 1 *ind*1 *sam*7 *nin*5])	Stratagene
*E. coli* KDZif1DZ	*ara*D (*gpt-lac*)5, *rps*L (Str^R^), Δ*spo*S3::*cat* (Cam^R^) [F’ *lac*I^*q*^ (Z321(-61) lacZYA*) Kan^R^]	Vallet-Gely et al., 2005
*E. coli* BW25113	F^–^, Δ*(araD-araB)567*, Δ*lacZ4787*(::rrnB-3), *λ-*, *rph-1*, Δ*(rhaD-rhaB)568*, *hsdR514*	Keio collection
*E. coli* BW25113 Δ*Lon* (JW0429-1)	F^–^, Δ*(araD-araB)567*, Δ*lacZ4787*(::rrnB-3), Δ*lon-725::kan*, *λ-*, *rph-1*, Δ*(rhaD-rhaB)568*, *hsdR514*	Keio collection
**Plasmids**	**Characteristics**	**References**
p15TV-L	Expression vector, T7 promoter with *lac* operator, Amp^R^	Structural Genomics Consortium, University of Toronto
p15TV-L_His.LotP	LotP gene cloned by ligation independent cloning in p15TV-L, Amp^R^	Loto et al., 2017
p15TV-L_His.LotP^R104A^	p15TV-L_His.LotP vector with the mutation R104A, Amp^R^	This work
p15TV-L_His.EK.LotP	His.EK.LotP gene cloned between the *Nco*I and *Xho*I sites of p15TV-L, Amp^R^	This work
p15TVL_His.EK.LotP^R104A^	p15TV-L_His.EK.LotP vector with the mutation R104A, Amp^R^	This work
p15TVL_His.LotP.FLAG	LotP.FLAG gene cloned by ligation independent cloning in p15TV-L, Amp^R^	This work
pCDF-1b	Expression vector, T7 promoter with *lac* operator, Spec^R^	Novagen
pCDF-1b_His.EK.LotP.FLAG	LotP.FLAG gene cloned between the *Bam*H and *Xho*I sites of pCDF-1b, Spec^R^	This work
pCDF-1b_His.EK.LotP	LotP gene cloned between the *Bam*H and *Xho*I sites of pCDF-1b, Spec^R^	This work
pCDF-1b_LotP.His	LotP.His gene cloned between the *Nco*I and *Xho*I sites of pCDF-1b, Spec^R^	This work
pCDF-1b_ΔNLotP.His	ΔNLotP.His gene cloned between the *Nco*I and *Xho*I sites of pCDF-1b, Spec^R^	This work
pCDF-1b_N-terminal(Lon)-LotP.His	LotP.His gene with the N-terminal of the Lon protease cloned between the *Nco*I and *Xho*I sites of pCDF-1b, Spec^R^	This work
pCDF-1b_N-terminal(Lon)-ΔNLotP.His	ΔNLotP.His gene with the N-terminal of the Lon protease cloned between the *Nco*I and *Xho*I sites of pCDF-1b, Spec^R^	This work
pCDF-1b_FLAG.MBP.C-terminalSulA	MBP with a FLAG tag in the N-terminal and the C-terminal of *E. coli* SulA gene in the C-terminal, cloned between the *Nco*I and *Sac*I sites of pCDF-1b, Spec^R^	This work
pBRGP-ω	Translational fusion vector, Amp^R^	Vallet-Gely et al., 2005
pBRGP-ω_LotP	LotP gene cloned between the *Nde*I and *Not*I sites of pBRGP- ω, Amp^R^	Loto et al., 2017
pBRGP-ω_Lon	Lon gene cloned between the *Nde*I and *Not*I sites of pBRGP- ω, Amp^R^	This work
pBRGP-ω_ΔNLon	ΔNLon gene cloned between the *Nde*I and *Not*I sites of pBRGP- ω, Amp^R^	This work
pBRGP-ω_LotP^R104A^	LotP^R104A^ gene cloned between the *Nde*I and *Not*I sites of pBRGP- ω, Amp^R^	This work
pACTR-AP-Zif	Translational fusion vector, Tet^R^	Vallet-Gely et al., 2005
pACTR-AP-Zif_LotP	LotP gene cloned between the *Nde*I and *Not*I sites of pACTR-AP-Zif, Tet^R^	Loto et al., 2017
pACTR-AP-Zif_Lon	Lon gene cloned between the *Nde*I and *Not*I sites of pACTR-AP-Zif, Tet^R^	This work
pACTR-AP-Zif_ΔNLon	ΔNLon gene cloned between the *Nde*I and *Not*I sites of pACTR-AP-Zif, Tet^R^	This work
pACTR-AP-Zif_LotP^R104A^	LotP^R104A^ gene cloned between the *Nde*I and *Not*I sites of pACTR-AP-Zif, Tet^R^	This work
pBAD24	Expression vector, arabinose inducible promoter, Amp^R^	Guzman et al., 1995
pBAD24_LotP	LotP gene cloned between the *Kpn*I and *Sal*I sites of pBAD24, Amp^R^	Loto et al., 2017
pBAD24_LotP^R104A^	LotP^R104A^ gene cloned between the *Kpn*I and *Sal*I sites of pBAD24, Amp^R^	This work
pCA24N	Expression vector, P_*T5–lac*_ promoter, Clm^R^	Hirotada Mori Lab
pCA24N_SulA	pCA24N plasmid carrying *E. coli* SulA, strain JW0941, Clm^R^	ASKA collection
pCA24N_Lon	pCA24N plasmid carrying *E. coli* Lon protease, strain JW0429, Clm^R^	ASKA collection

### Cloning and Protein Purification

Standard methods were used for restriction enzyme digestion and molecular recombination. *E. coli* cell transformation was done as previously described (Sambrook et al., 1989). Plasmids were isolated using the QIAprep^®^ Spin Miniprep Kit (Qiagen, Valencia, CA, United States), and PCR products were purified using QIAquick^®^ purification kits (Qiagen). Proteins were expressed with a His_6X_-tag in the strain *E. coli* BL21 (DE3) and purified as previously described (Loto et al., 2017).

### LotP Site-Directed Mutagenesis

CLIBASIA_03135 was previously cloned in the plasmid p15TV-L (GenBank accession EF456736). Site-directed mutagenesis was performed into this clone/construction; the sequence of the primers used for each mutation is listed in Supplementary Table 1. The methods used for mutagenesis were described by Edelheit et al. (2009).

### Cloning of N-Terminal and C-Terminal Modified LotP

N- and C-terminal modified LotP constructs were cloned into the plasmid pCDF-1b (Novagen, Merck KGaA, Darmstadt, Germany) and p15TV-L. The sequences were amplified by PCR using the primers listed in Supplementary Table 1 and cloned in the vector using the restriction sites introduced within each primer. The coding sequence for the His.EK cleavage site was added at the N-terminal of LotP by cloning it into the plasmid pCDF-1b. FLAG- or His-tag was introduced to the N-terminal or C-terminal by modifying the primers used in PCR amplification. N-terminal(Lon)-LotP.His and N-terminal(Lon)-ΔNLotP.His were amplified using a two-step PCR with Fw_N1LotP or Fw_N2LotP as forward primer in the first amplification and Fw_N3LotP_*Nco*I in the second amplification. The reverse primer, Rv_LotP_HisStop_*Xho*I, was the same in all three PCRs.

Size-exclusion chromatography was performed as described previously (Loto et al., 2017). Superose 12 10/300 chromatography column (GE Healthcare, Chicago, IL, United States) was calibrated with cytochrome *c* from horse heart (12.4 kDa), carbonic anhydrase from bovine erythrocytes (29 kDa), albumin from bovine serum (66 kDa), alcohol dehydrogenase from yeast (150 kDa), and β-amylase from sweet potato (200 kDa). In the case of Superose 6 10/300 chromatography column (GE), apoferritin from equine spleen (443 kDa), and thyroglobulin from bovine thyroid (669 kDa) were added to the standard mix. Each construction was analyzed with samples from at least two different purifications process, and each sample was run at least three times to confirm the profile and elution volumes.

### LotP Dimerization

The cross-linking assay was done as previously described with minor modifications (Lee et al., 2004). Briefly, purified LotP protein (2 μg) was incubated with 2% formaldehyde at 25°C, and the reaction was stopped after 10 or 60 min with the addition of glycine to a final concentration of 0.125 M. Cross-linked products were analyzed by sodium dodecyl sulfate–polyacrylamide gel electrophoresis (SDS-PAGE) (10%).

### Circular Dichroism

Purified LotP protein (∼0.2 mg/ml) was dialyzed with a solution of 5 mM of MgSO_4_, 36.4 mM of Na_2_HPO_4_, and 3.6 mM of NaH_2_PO_4_. UV circular dichroism (CD) spectra (190–260 nm) was recorded at 25°C on a Circular Dichroism Spectrometer Model 400 (Biomedical, Inc., Lakewood, NJ, United States). We used a 1-mm path length cell at 1-nm step resolution and 10 nm/min scan speed. All spectra were corrected by subtracting the control measurement of dialysis buffer and normalized by the mean residue weight (MRW) of the proteins, path length (P, cm), and protein concentration (C, mg ml^–1^). The final spectra were expressed in Δε (M^–1^ cm^–1^), where Δε = θ ^∗^ (0.1 ^∗^ MRW)/(P ^∗^ C ^∗^ 3,298). Machine units (θ) were obtained in millidegrees from the spectrometer.

### Limited Proteolysis Assay

Limited proteolysis of purified LotP proteins was done as described by Cellini et al. (2010), with minor modifications. Briefly, purified proteins were incubated with proteinase K (ratio of 300:1) at 25°C. Aliquots were taken at 0, 5, 15, 45, 90, and 240 min. The reaction was stopped with phenylmethylsulfonyl fluoride (PMSF), and the samples analyzed by SDS-PAGE (12.5%).

### Two-Hybrid Assay

The protein–protein interaction between LotP, LotP mutants, and the Lon protease (CLIBASIA_00775) was assessed by cloning the genes and encoding each protein into a bacteria two-hybrid system. Briefly, the sequence of Lon protease and the modified versions of LotP from ‘*Ca.* L. asiaticus’ were amplified by PCR using the primers listed in Supplementary Table 1. Each sequence was fused to the ω subunit of the RNAP by cloning the fragment into the *Nde*I and *Not*I sites of the pBRGP-ω plasmid. The same genes were cloned and fused to the zinc finger DNA-binding protein of the murine Zif268 transcription factor using same restriction sites for the plasmid pACTR-AP-Zif. The recombinant clones were expressed into the reporter *E. coli* strain KDZif1DZ. β-Galactosidase activity was measured as previously described (Loto et al., 2017). Each assay was performed in biological and technical triplicates—induced with 20 μM of IPTG.

### *Escherichia coli* Growth Kinetics Assays

Two *E. coli* strains were used in the reported growth curves: BW25113 as the wild type (wt) and BW25113 with a knockout of the *lon* gene (Δ*Lon*, strain JW0429). The strains were transformed with pBAD24_LotP and pCA24N_SulA (from strain JW0941, ASKA collection) as well as pBAD24_LotP^R104A^, which was amplified and cloned using primers listed in Supplementary Table 1. The empty pBAD24 vector was used as a control. Growth curves were carried out using M9 media with the addition of 0.1 mM of CaCl_2_, 2 mM of MgCl_2_, and 0.2% v/v glycerol as the sole carbon source. Cells were incubated at 37°C and 200 rpm. Individual flasks were inoculated with aliquots obtained from overnight cultures grown in LB broth at starting OD_600 *nm*_ = 0.05. The induction of gene expression was done at the beginning of each assay with L-arabinose (0.001–0.2%) for assays using the pBAD24 plasmid. IPTG (100 μM) was used for pCA24N plasmid. Cellular growth was monitored at OD_600_; all assays were done in triplicate.

### Western Blotting Analysis

Samples analyzed with SDS-PAGE (12.5%) were transferred onto a 0.45 μm of polyvinylidene difluoride (PVDF) membrane (Bio-Rad, Hercules, CA, United States) using a semi-dry blotting unit (Fisher Scientific, Hampton, NH, United States) at 450 mA for 40 min. The membranes were treated as described before (Loto et al., 2017). Primary antibodies used were rabbit anti-LotP, rabbit anti-FLAG (1:5,000, Sigma-Aldrich Corp., St. Louis, MO, United States) and mouse anti-His (1:3,000, Sigma-Aldrich). The secondary antibodies conjugated to horseradish peroxidase for chemiluminescence detection were anti-rabbit (1:20,000, Sigma-Aldrich) and anti-mouse (1:10,000, Sigma-Aldrich). Horseradish peroxidase (HRP) activity was detected using Amersham ECL^TM^ Western Blotting Detection Reagents (GE Healthcare, Pittsburgh, PA, United States). The bands in the membrane were visualized using the automatic imager FluorChem R (ProteinSimple, San Jose, CA, United States), and the band intensity was quantified by ImageJ software (Schneider et al., 2012).

### Lon Protease Activity Assays *in vivo*

*Escherichia coli* strain BL21 (DE3) was transformed with p15TV-L_His.LotP, p15TV-L_His.EK.LotP, pCA24N_Lon (pCA24N plasmid carrying *E. coli* Lon protease, strain JW0429 ASKA collection), and pCDF-1b_FLAG.MBP.C-terminalSulA; empty plasmids were used as controls. p15TV-L_His.EK.LotP was cloned from the digestion of pCDF-1b_His.EK.LotP using *Nco*I and *Xho*I restriction enzymes and a ligation in p15TV-L digested with the same restriction enzymes. pCDF-1b_FLAG.MBP.C-terminalSulA was cloned using a PCR product obtained from a two-step PCR amplification digested with *Nco*I and *Sac*I. The SulA fusion was expressed from the pCDF1-b in *E. coli* BL21(DE3), a protease negative strain. The *E. coli* Lon protease was expressed from the vector pCA24N, while His.LotP and His.EK.LotP were expressed from p15TV-L. Once the different combinations of the proteins were induced, the amount of remaining proteins for each case/combination was analyzed by Western blotting.

The *in vivo* degradation assay was carried out as described previously (Ishii et al., 2000). Briefly, flasks containing LB media were inoculated with aliquots from overnight cultures at OD_600 *nm*_ = 0.025. Genes were induced with 0.05 mM of IPTG (3 h of culture); and protein expression was stopped with tetracycline 30 min after induction. The samples obtained were treated with PMSF to prevent further proteolysis. MBP.SulA degradation was monitored by Western blotting using anti-FLAG antibodies.

### Plant Material and LotP Infiltration

*Citrus sinensis* cv. Valencia on Kuharski rootstock plants were obtained from Phillip Rucks Citrus Nursery (Frostproof, FL, United States). Plants were grown in a greenhouse with a relative humidity of 74% and a controlled temperature around 27°C. Plants (6–8 months old) were moved to an indoor growth chamber 2 weeks before infiltrations. The chamber was kept at 24°C with 14 h of light and 10 h of dark per day. The leaves were cleaned with 10% bleach followed by water prior to infiltrations. Purified protein, diluted to the working concentration with 10 mM of MgCl_2_, was used to infiltrate citrus leaves. Identical buffer, with no protein, was infiltrated as a control in each leaf.

### Sample Preparation for TMT 11-Plex

For proteomic analysis, six plants were used, two for each treatment group. Fifteen leaves per treatment group (His.LotP and His.EK.LotP.FLAG) were fully infiltrated in all lobes at a concentration of 8 μM. Biological replicates (one leaf per tree) were taken at 1, 2, and 3 dpi (days post infiltration) for a total of 18 leaves. Once collected, leaves were frozen immediately in liquid nitrogen and subsequently placed at –80°C until further use. Phenol extracted proteins were precipitated and were further washed twice with cold 80% acetone (stored at –20°C), incubating overnight each time. The final pellet was re-suspended in 500 μl of ITRAQ protein buffer (0.4 M of urea, 12.5 mM of TEAB, 1% Triton X-100, and 0.05% SDS). Precipitated proteins were further used for acetone precipitation. Protein concentrations were determined using the Bradford assay and run on a 12.5% SDS-PAGE gel to ensure protein extraction quality.

### Protein Sample Preparation, Digestion, and TMT Labeling

Proteins were dissolved in protein buffer [8 M of urea, 50 mM of Tris-HCl, pH 8.0, 50 mM of triethylammonium bicarbonate, 0.1% SDS (w/v), 1% Triton-100 (w/v), 1 mM of PMSF, 10 μg/ml of leupeptin, and 1% phosphatase inhibitor cocktail 2 and 3 (v/v)]. For each sample, a total of 200 μg of protein was reduced with 10 mM of tris(2-carboxyethyl)phosphine, alkylated with 20 mM of iodoacetamide, trypsin-digested (w/w for enzyme:sample = 1:100), and labeled according to the manufacturer’s instructions (Thermo Scientific, Rockford, IL, United States). Control samples with three different day points of Days 1, 2, and 3 were labeled with TMT tags 126, 127N, and 127C, respectively, and the LotP samples were labeled with TMT tags 128N, 128C, and 129N, respectively. The corresponding FLAG samples were labeled with 129C, 130N, and 130C, respectively. To increase the signal, mixed samples of LotP and FLAG were labeled with 131N, and mixed samples of controls were labeled with 131C, which works as an internal standard. Therefore, all experiments were performed with at least biological duplicates in time-point per group. Labeled peptides were desalted with C18-solid phase extraction and dissolved in strong cation exchange (SCX) solvent A [25% (v/v) acetonitrile, 10 mM of ammonium formate, and 0.1% (v/v) formic acid, pH 2.8].

### Strong Cation Exchange Fractionation and Reverse-Phase LC-MS/MS

The peptides were fractionated using an Agilent HPLC 1260 with a polysulfoethyl A column (2.1 × 100 mm, 5 μm, 300 Å; PolyLC, Columbia, MD, United States). Peptides were eluted with a linear gradient of 0–20% solvent B [25% (v/v) acetonitrile and 500 mM of ammonium formate, pH 6.8] over 50 min followed by ramping up to 100% solvent B in 5 min.

A hybrid quadrupole Orbitrap (Q Exactive Plus) MS system (Thermo Fisher Scientific, Bremen, Germany) was used with high-energy collision dissociation (HCD) in each MS and MS/MS cycle. The MS system was interfaced with an automated Easy-nLC 1000 system (Thermo Fisher Scientific, Bremen, Germany). Each sample fraction was loaded onto an Acclaim Pepmap 100 pre-column (20 mm × 75 μm; 3 μm-C18) and separated on a PepMap RSLC analytical column (250 mm × 75 μm; 2 μm-C18) at a flow rate at 350 nl/min during a linear gradient from solvent A [0.1% formic acid (v/v)] to 30% solvent B [0.1% formic acid (v/v) and 99.9% acetonitrile (v/v)] for 95 min, to 98% solvent B for 15 min, and hold 98% solvent B for additional 30 min. Full MS scans were acquired in the Orbitrap mass analyzer over *m*/*z* 400–2,000 range with resolution 70,000 at 200 *m*/*z*. The top 10 most intense peaks with charge state ≥ 2 were fragmented in the collision cell with a normalized collision energy of 28. The quadrupole isolation window was 0.7 *m*/*z*. The maximum ion injection time for both the survey scan and the MS/MS scan was 250 ms, and the ion target values were set to 3^e6^ and 1^e6^, respectively. Selected sequenced ions were dynamically excluded for 60 s.

### Proteomics Data Analysis

The raw MS/MS data files were processed by a thorough database searching approach considering biological modification and amino acid substitution against Uniprot Citrus database (downloaded on June 6, 2019; 196,268 entries) using the Proteome Discoverer v2.3 (Thermo Fisher Scientific), with the SEQUEST algorithm (Eng et al., 1994). The following parameters were used for all the searching: peptide tolerance at 10 ppm, tandem MS tolerance at ±0.02 Da, peptide charges of 2+ to 5+, trypsin as the enzyme, allowing one missed cleavage, TMT label and carbamidomethyl (C) as fixed modifications, acetylation (*n*-terminus), 2-hydroxyisobutyrylation (K), loss of methionine (n-terminus), oxidation (M), and phosphorylation (S, T, and Y) as variable modifications. For peptide confidence, we adopted the following cutoff values of Xcorr that are commonly used for the SEQUEST algorithm (Nesvizhskii et al., 2003): 2.31 for 2+, 2.41 for 3+, and 2.6 for 4+ and 5+ peptides. The false discovery rate (FDR) was calculated using the Percolator algorithm in the Proteome Discoverer workflow based on the search results against a decoy database and was set at 1% FDR. Duplicates were grouped, and statistical analyses evaluated as the quantitative ratio between each control and treatment per day. Furthermore, proteins identified which contained at least two peptides with a *p*-value of <0.05 and log2 fold ratio greater or less than 0.5.

Mercator was used for a genome-wide functional annotation of the UNIPROT Citrus Database (June 6, 2019), creating MapMan Bin codes. Pathway analysis was curated using PageMan that is fully integrated into the MapMan application.

The mass spectrometry proteomics data have been deposited to the ProteomeXchange Consortium via the PRIDE (Deutsch et al., 2017) partner repository with the dataset identifier PXD020912 and doi: 10.6019/PXD020912.

### Plant Photochemistry Measurements

Maximum quantum yields of photosystem II (PSII) were estimated from chlorophyll fluorescence measurements using the Opti-Sciences PSK (Opti-Sciences, Hudson, NH, United States). Dark adaption time was determined by checking the value of the maximum PSII quantum yield, F_*v*_/F_*m*_, of citrus leaves at different dark-adapted times. It was determined that 15 min of dark adaptation was appropriate for a stable F_*v*_/F_*m*_ of 0.80 with fluorometer settings as follows: saturation pulse intensity of 1, saturation pulse width of 1, modulated light intensity of 2, and gain of 6. Chlorophyll fluorescence was measured at the middle part of the abaxial side of the third lobe from the top of the leaf with the *in situ* portable fluorometer. The minimum fluorescence in dark-adapted state (F_0_) was measured after 15 min dark adaptation. Measurements were taken daily. Based on the measured fluorescence signals, the variable fluorescence in dark-adapted state (F_*v*_ = F_*m*_ – F_0_) can also be obtained.

## Results

### Assessing the Contribution of Conserved Amino Acids in LotP Folding

**FIGURE 1:**
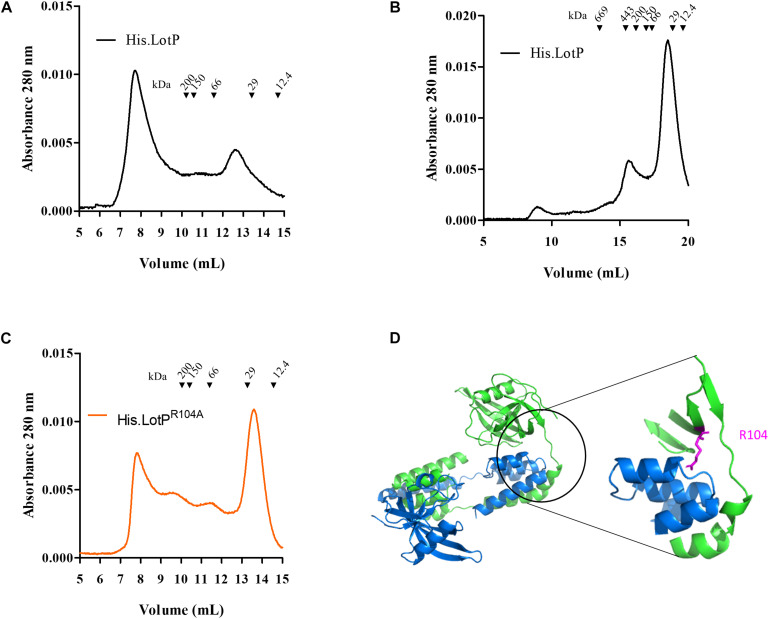
Equilibrium of LotP dimers—oligomers and the contribution of conserved amino acids in LotP folding. Representative chromatograms of purified His.LotP analyzed by size-exclusion chromatography using a Superose 12 column **(A)** and Superose 6 column **(B)**. **(C)** Representative chromatograms of purified and His.LotP^R104A^ analyzed by size-exclusion chromatography using a Superose 12 column. The purified proteins were separated according to its native molecular size with the varying oligomers. Elutions were monitored continuously at 280 nm, and the graphics represent the signal at the different elution volumes. **(D)** LotP dimer structure modeled with GalaxyWEB sever using the crystal structure of *Bacillus subtilis* Lon N-terminal domain (PDB: 3M65A) as template. The leading model is a dimer with a prediction score of 305.854 and structural similarity of 0.9547. The inset of the dimer structure shows the mutated residue R104 in pink.

We had previously reported that stabilized soluble LotP, carrying a histidine tag and a TEV cleavage site in its N-terminal (His.LotP), eluted from a Superose 12 column as a dimer of 44 kDa (∼12.8 ml) after purification. A deeper analysis of our samples showed a minimal fraction of higher-molecular-weight complexes, with a peak out of the resolution of the column (∼7.6 ml) (Figure 1A). In order to fully evaluate the importance of the high-molecular-weight complexes of His.LotP, we have analyzed native and site-directed mutants of newly purified samples. We had observed high-molecular-weight complexes using a Superose 6 column (∼300 kDa, ∼ 15.72 ml) corresponding to structures of 12 monomeric subunits (Figure 1B). The Superose 6 column has better resolution for protein of higher molecular weight. Aimed to identify critical residues holding these structures together, the native molecular weight of a library of site-directed mutants (18 in total) was also analyzed. The mutation sites were selected based on amino acid conservancy among LotP homologues (Supplementary Figure 1). Noteworthy, the single alanine replacement LotP^R104A^ shifted a large fraction of the multimeric units observed to monomers of 24 kDa (∼13.7 ml) (Figure 1C). The spatial location of this relevant amino acid in the predicted protein structure is shown in Figure 1D. The model analysis suggests that this amino acid is in a critical position connecting the two main domains of the protein.

### LotP N-Terminal Domain May Contribute to the Formation of Multimers

**FIGURE 2:**
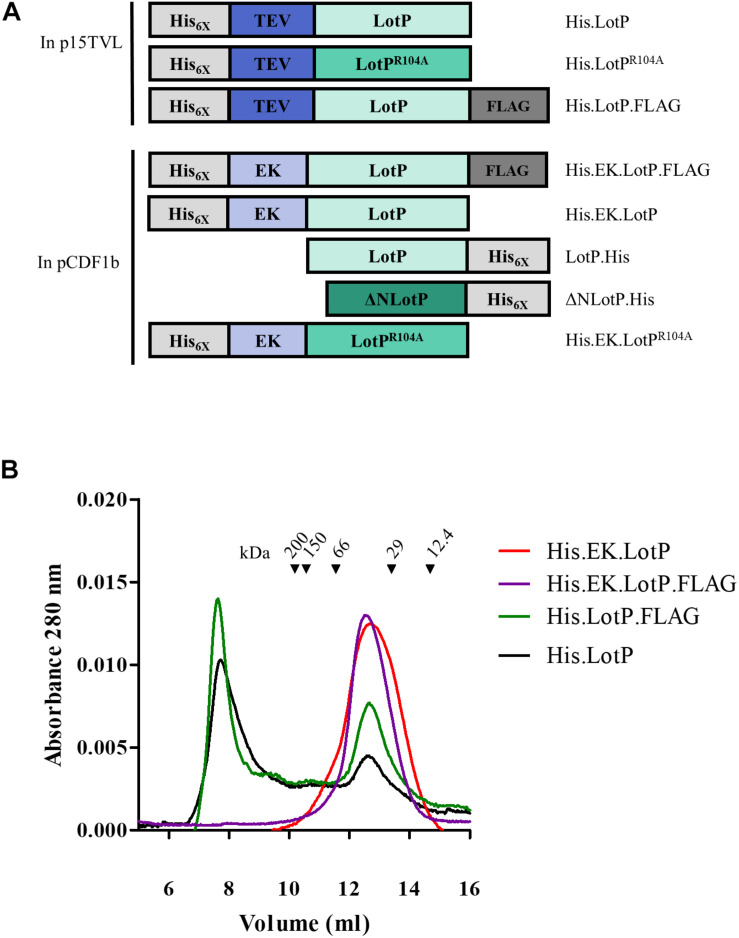
Modifications to LotP and its effect on dimerization. Schematic representation of different LotP constructions. **(A)** Protein tags (His_6X_ and FLAG) are shown in shades of gray, cleavage sites (TEV and EK) in shades of blue, and LotP protein constructs (LotP, LotP^R104A^, and ΔNLotP) in shades of green. **(B)** Representative chromatogram of purified His.EK.LotP (red), His.EK.LotP.FLAG (purple), His.LotP.FLAG (green), and His.LotP (black) analyzed by size-exclusion chromatography using a Superose 12 column. The purified proteins were separate according to its native molecular size. The elutions were monitored continuously at 280 nm, and the graphic shows the signal at the different elution volumes.

The results obtained with the single LotP^R104A^ mutant prompted us to evaluate the contribution of the N- and C-terminal domains of LotP on the molecule oligomerization. Understanding the stability of these large-molecular-weight complexes will help us to better evaluate the biological role of the protein in the pathosystem. The two domains are well defined and separated by a flexible alpha helix of 23–28 amino acids (Figure 1D). To address the relevance of each domain in oligomerization, the recombinant proteins were modified as described in Figure 2A. Adding a FLAG (His.LotP.FLAG) or a histidine tag (LotP.His) to the carboxy terminal showed an identical oligomerization profile to that of His.LotP (Figure 2B and Supplementary Figure 2). Thus, we concluded that modifications to the C-terminal have no effect on the formation of multimers.

To address the importance of the N-terminal domain in the formation of protein multimers, the TEV site was replaced by an enterokinase (EK) cleavage site. The modified proteins, named His.EK.LotP.FLAG and His.EK.LotP, eluted as a unique peak of 44 kDa (12.7 ml) from the Superose 12 column corresponding to dimers in solution (Figure 2B).

**FIGURE 3:**
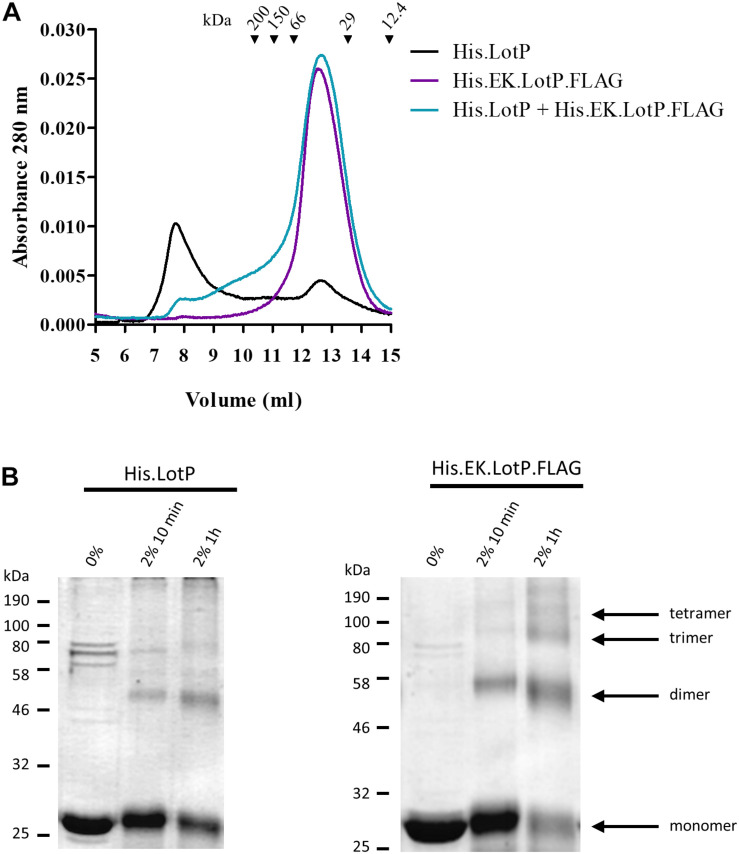
Analysis of LotP multimers. **(A)** Representative chromatograms of purified His.LotP (black), His.EK.LotP.FLAG (purple), and His.LotP + His.EK.LotP.FLAG (teal) analyzed by size-exclusion chromatography using a Superose 12 column. The purified proteins were separate according to its native molecular size. The elutions were monitored continuously at 280 nm, and the graphic shows the signal at the different elution volumes. **(B)** Sodium dodecyl sulfate–polyacrylamide gel electrophoresis (SDS-PAGE) of samples of purified His.LotP and His.EK.LotP.FLAG proteins cross-linked with 2% formaldehyde for either 10 min or 1 h and a control with no formaldehyde. The assay was done in triplicates, while a representative gel is shown in the figure. A total of 2 μg of protein was used per lane.

Furthermore, we hypothesized that LotP has a dynamic behavior in solution where several oligomeric forms coexisting. To verify our hypothesis, His.LotP was mixed with a two-fold excess of His.EK.LotP.FLAG, and the samples were analyzed by size-exclusion chromatography. The results indicated that His.LotP interacts with His.EK.LotP.FLAG, shifting most of the multimeric complexes to a uniform population of dimers in solution (Figure 3A). The total amount of protein in the mixture remained constant, as no protein precipitation was observed after centrifugation. These observations suggested that the synthetic heterodimer remained in solution.

A time course cross-linking assay using the purified proteins, separately, indicated that both His.LotP and His.EK.LotP.FLAG can form multimers of approximately 25, 50, 75, and 100 kDa or of even higher molecular weights (Figure 3B). Still, after 1 h in solution, the proportion of dimers was significantly higher in the case of His.EK.LotP.FLAG, indicating either a higher stability of the dimers or slower rate of multimers formation for the recombinant protein. In summary, the results suggested that LotP has a dynamic behavior in solution—going from dimers to a steady formation of multimers.

**FIGURE 4:**
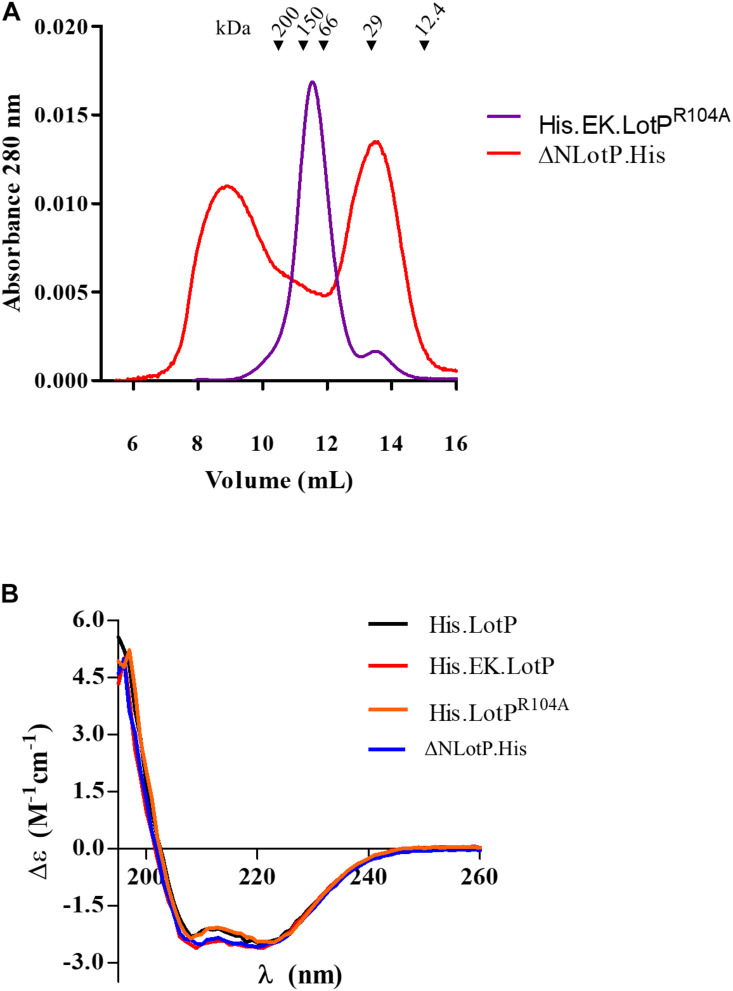
Modifications to LotP and its effect on structure and dimerization. **(A)** Representative chromatogram of purified His.EK.LotP^R104A^ (red) and ΔNLotP.His (blue) analyzed by size-exclusion chromatography using a Superose 12 column. The purified proteins were separate according to its native molecular size. The elutions were monitored continuously at 280 nm, and the graphic shows the signal at the different elution volumes. **(B)** Purified His.LotP (black), His.EK.LotP (red), His.LotP^R104A^ (orange), and ΔNLotP.His (blue) proteins analyzed by far UV circular dichroism spectra. Each curve is an average of three scans, normalized by the mean residue weight of each proteins, path length, and protein concentration.

To further understand the significance of multimer formation, we introduced additional modifications to the N-terminal domain of LotP (Figure 2A). Deletion of 13 amino acids in the amino terminal (ΔNLotP.His) and further comparative analysis with size-exclusion chromatography revealed that the deletion did not abolish multimer formation but shifted a large fraction of proteins to monomeric subunits (Figure 4A). When the His.EK site was inserted in the N-terminal of LotP^R104A^ (His.EK.LotP^R104A^), only complexes of ∼112 kDa (hexamers) were observed (Figure 4A). These results suggest that changes in the overall scaffold of the N-terminal sequence may compensate for the effects of the punctual mutation. Lacking the structure of this protein, we are unable to elucidate this intriguing effect using closely related models.

Several LotP constructs were analyzed for structural changes using CD and a limited proteolysis assay with proteinase K. The far-UV CD spectrum representing the ratio of α-helices and β-sheets present in the secondary structure shows no apparent changes in any of the analyzed proteins (Figure 4B). Proteinase K assays demonstrated that mutant R104A suffered less degradation than other LotP variants, suggesting small changes in protein structure, making the protein less prone to degradation (Supplementary Figure 3).

The formation of multimers suggested that this protein could form higher-level structures like the active conformation of many proteolytic complexes. The structural similarities of the main protein scaffold with the substrate binding domain of the Lon protease prompted us to evaluate protein interactions between LotP and the Lon protease.

### The Lon Protease, a New Member of the LotP Interactome

**FIGURE 5:**
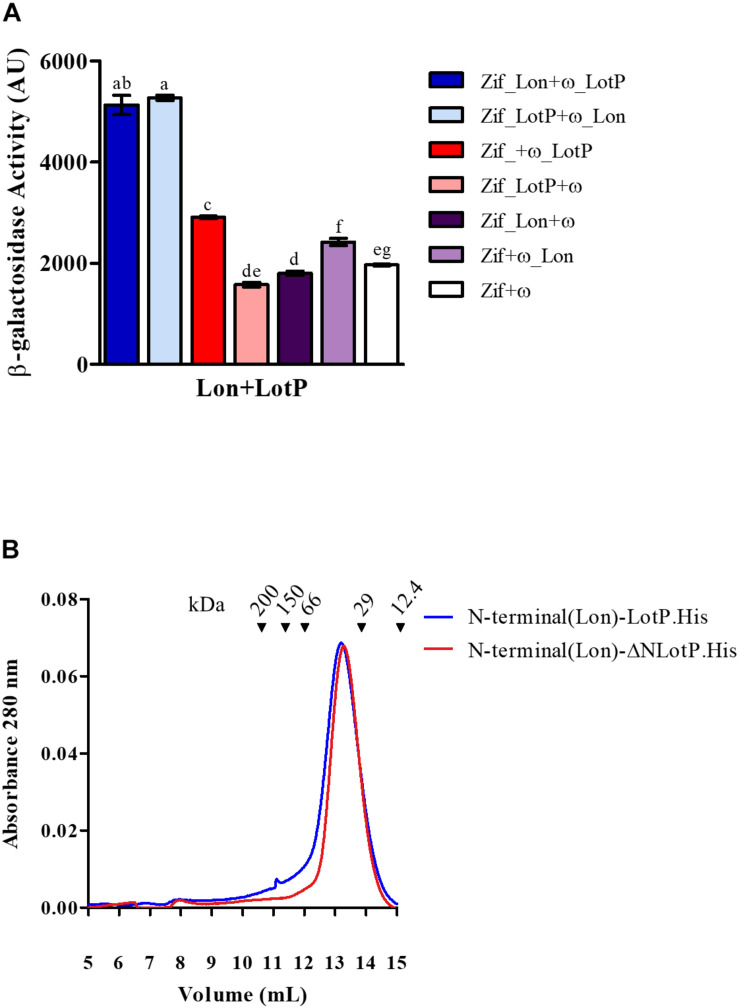
Lon-LotP interactions and analysis of Lon’s N-terminal. **(A)** Interaction between LotP and Lon protease. β-Galactosidase activity was determined using *Escherichia coli* strain KDZif1DZ as a reporter strain, transformed with the different construction of the plasmid pACTR-AP-Zif (Zif) and pBRPG-ω (ω). Assays were performed at different stages of exponential growth (OD_600_ = 0.3, 0.6, 0.9), while only OD_600_ = 0.9 is shown. β-Galactosidase activity is shown as the average ± SD in arbitrary units (AU) from biological triplicates. Variance was calculated by one-way ANOVA with a Tukey’s *post hoc* test, *p* = 0.05. Significant difference between two samples is denoted by different letters. **(B)** Purified N-terminal(Lon).LotP.His (blue) and N-terminal(Lon)-ΔNLotP.His (red) proteins analyzed by size-exclusion chromatography using a Superose 12 column. The purified proteins were separate according to its native molecular size. The elutions were monitored continuously at 280 nm, and the graphic shows the signal at the different elution volumes.

It was previously reported that LotP from ‘*Ca.* L. asiaticus’ interacts with different components of the proteostasis network found in *E. coli*, *Sinorhizobium meliloti*, and ‘*Ca*. L. asiaticus’ (Loto et al., 2017). The Lon protease, similar in structure to LotP, belongs to this same big group of proteins (Cho et al., 2015). Given this connection, the ‘*Ca.* L. asiaticus’ Lon protease was cloned, and its interaction with LotP was evaluated using a bacterial two-hybrid system (Vallet-Gely et al., 2005). A significant interaction between Lon protease and LotP was observed (Figure 5A).

After the determination of this interaction, we studied the importance of the N-terminal of these proteins, as well as residue R104 of LotP, in this interaction. When comparing the Lon protease of ‘*Ca.* L. asiaticus’ with other Lon proteases, there is a distinct extension to the N-terminal (Supplementary Figure 4). The N-terminal of this Lon protease contains several additional charged amino acids (EDESKDR), thereby showing sequence similarity to the EK sequence used in the LotP constructs described above. We hypothesized that this sequence could regulate the interaction between these two proteins. We constructed two chimeras by adding the N-terminal of the Lon protease (20 amino acids) to LotP.His and ΔNLotP.His, creating N-terminal(Lon)-LotP.His and N-terminal(Lon)-ΔNLotP.His, respectively. Surprisingly, both proteins eluted as dimers when analyzed by size-exclusion chromatography (Figure 5B). These results were comparable with those described for the construct His.EK.LotP and His.EK.LotP.FLAG (Figure 2B)—all with important modifications in the N-terminal. These results confirmed the critical contribution of the N-terminal domain sequence in oligomerization.

**FIGURE 6:**
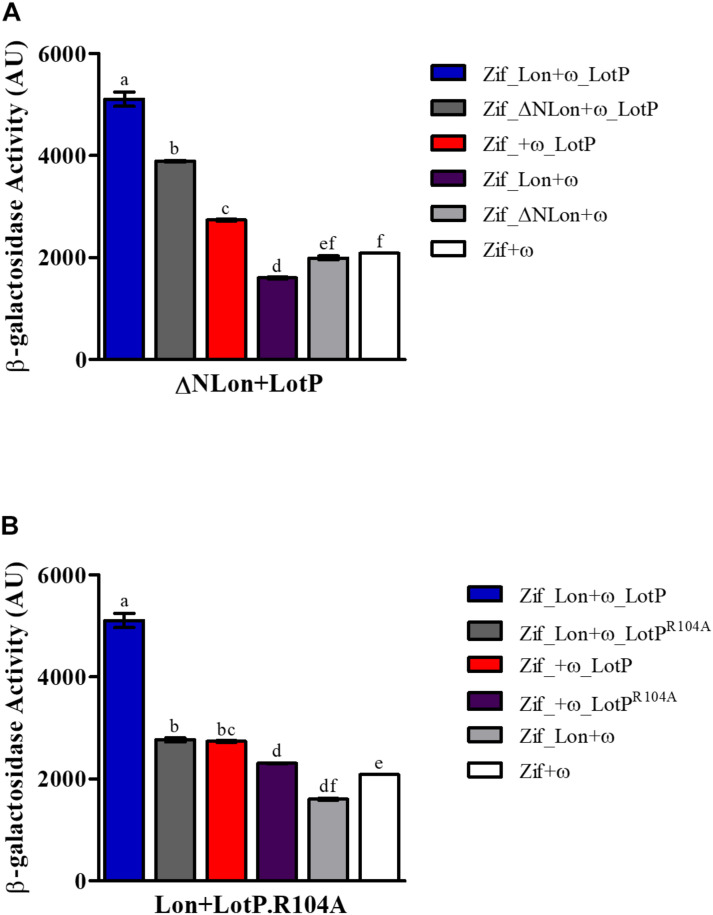
Structural factors that affect Lon–LotP interactions. β-Galactosidase activity was determined using *Escherichia coli* strain KDZif1DZ as a reporter strain, transformed with the different construction of the plasmid pACTR-AP-Zif (Zif) and pBRPG-ω (ω). Assays were performed at different stages of exponential growth (OD_600_ = 0.3, 0.6, 0.9), while only OD_600_ = 0.9 is shown. **(A)** Interaction between LotP and ΔNLon. **(B)** Interaction between LotP^R104A^ and Lon protease. β-Galactosidase activity is shown as the average ± SD in arbitrary units (AU) from biological triplicates. Variance was calculated by one-way ANOVA with a Tukey’s *post hoc* test, *p* = 0.05. Significant difference between two samples is denoted by different letters.

To assess if the N-terminal region mediates LotP–Lon interaction, we designed a new two-hybrid assay. The experiment was carried out using LotP and a Lon protease missing the first 23 amino acids of its N-terminal (ΔNLon). In this assay, the ω subunit was fused to LotP, while the Zif domain was fused to either Lon protease constructs, as this orientation provides optimal interfacing with LotP. The interaction of ΔNLon with LotP decreased by 50% when compared with interactions between the control Lon and LotP (Figure 6A). These results clearly demonstrate the importance of the Lon N-terminal sequence for the interaction between these two proteins. Interestingly, when mutant LotP^R104A^ was used in the same two-hybrid analysis, the β-galactosidase activity was arrested (Figure 6B).

### LotP Modulates Lon Protease Activity in *Escherichia coli*

**FIGURE 7:**
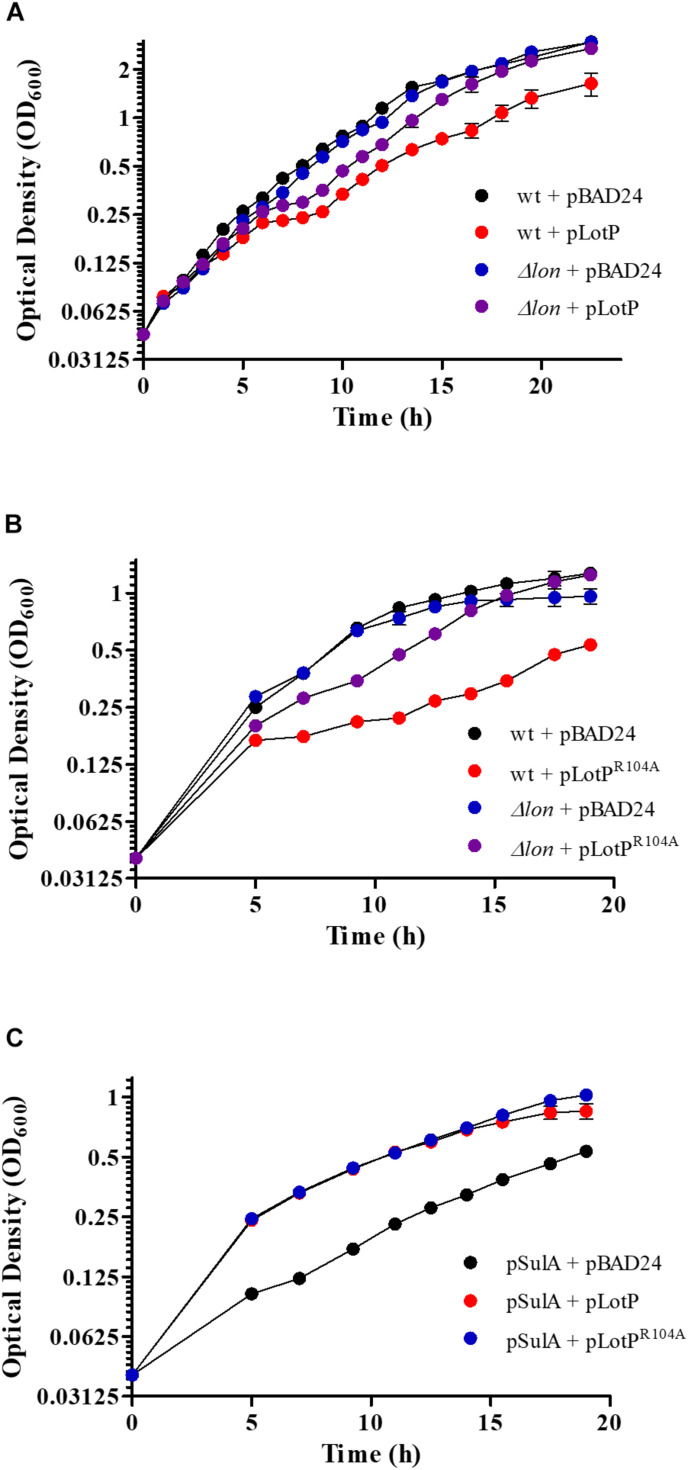
Effect of LotP on the growth of *Escherichia coli* wild type (wt) and Δ*lon*. Growth curves of wt and Δ*lon E. coli* overexpressing LotP constructs LotP and LotP^R104A^. Strains transformed with the pBAD24 vector were used as a control. Growth curve data represent averages ± SD (*n* = 3). **(A)** wt + pBAD24 (black), wt + pLotP (red), Δ*lon* + pBAD24 (blue), and Δ*lon* + pLotP (purple). **(B)** wt + pBAD24 (black), wt + pLotP^R104A^ (red), Δ*lon* + pBAD24 (blue), and Δ*lon* + pLotP^R104A^ (purple). Cultures were grown in M9 media with the addition of Ca^2+^, Mg^2+^, and glycerol; 0.2% L-arabinose was added for pBAD24 induction. **(C)** Growth curves of wt *E. coli* overexpressing SulA, LotP, and LotP^R104A^. Cultures were grown in M9 media with the addition of Ca^2+^, Mg^2+^, and glycerol. A low concentration of L-arabinose (0.001%) was added to induce the expression of pBAD24, and 100 μM of IPTG was added for SulA expression. pSulA + pBAD24 (black), pSulA + pLotP (red), and pSulA + pLotP^R104A^ (blue). Growth curve data represent averages ± SD (*n* = 3).

To acquire *in vivo* evidence in support of the molecular mechanisms described above, we designed a series of assays using the *E. coli* Lon protease as a central interaction target. As expected, LotP expression in a wt *E. coli* strain slightly affects cellular growth. Interestingly, this effect was not observed when LotP was expressed in a Δ*lon* isogenic strain (Figure 7A). When LotP^*R104A*^ was expressed, it affected the growth of the wt strain but did not affect the growth of the Δ*lon* mutant strain (Figure 7B). A Western blotting analysis indicated comparable protein abundance in all strains analyzed, validating the results observed (Supplementary Figures 5A,B). These results suggest that the presence of LotP may boost the activity of the Lon protease with deleterious effects in the cell physiology. It has been reported that high amounts of Lon protease negatively affect the growth of *E. coli* (Sonezaki et al., 1994).

To verify our hypothesis, we performed several assays using SulA, a well-known Lon protease target. This protein is a member of the SOS regulon, and its expression is triggered by DNA-damaging agents (Simmons et al., 2008). Once expressed, it inhibits bacterial septation by binding to FtsZ, stagnating the cellular growth. However, SulA is quickly degraded by the Lon protease, allowing for immediate resumption of division (Ishii et al., 2000). SulA was expressed from the plasmid pCA24N, while either LotP or LotP^R104A^ were expressed using pBAD24. After SulA expression was induced with 100 μM of IPTG, the growth of the *E. coli* wt strain was significantly affected. In the absence of the Lon protease (Δ*lon* mutants), the overexpression of SulA completely arrested the cellular growth. The wt growth was partially restored when LotP or LotP^R104A^ was induced (Figure 7C). A Western blotting revealed that SulA was only visualized when expressed in absence of LotP or LotP^R104A^ (Supplementary Figure 5C). These results suggest enhanced Lon protease activity when LotP is expressed. Additional assays are necessary to elucidate the results observed with the mutant protein.

### LotP Enhances Lon Protease Activity

**FIGURE 8:**
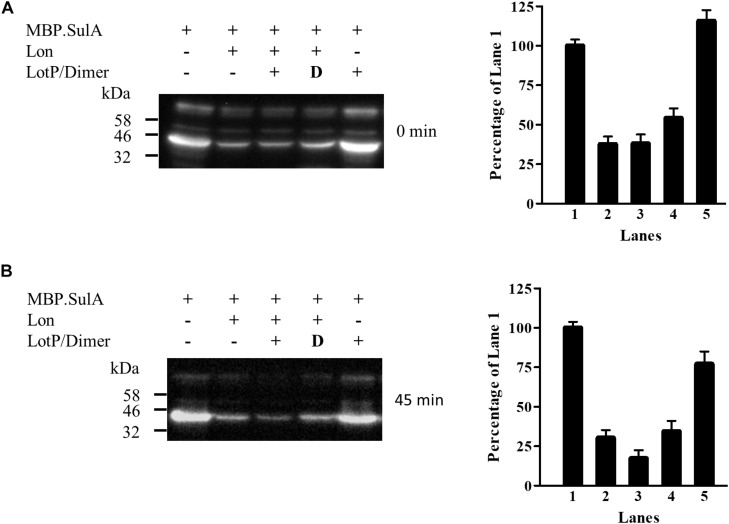
LotP increases the degradation of MBP.SulA in *Escherichia coli*. Western blotting using anti-FLAG antibodies of samples taken from *E. coli* overexpressing His.LotP, His.EK.LotP, Lon, and MBP.SulA (MBP.SulA has a FLAG tag in the N-terminal). After 30-min induction antibiotics was added to stop protein synthesis. Degradation was followed at 0 min **(A)** and 45 min **(B)** post incubation and phenylmethylsulfonyl fluoride (PMSF) added to halt degradation. The assay was done in duplicate, while a representative figure is shown in the left panels. The right panels show the quantification of the Western blotting bands. Columns were normalized to lane 1.

To analyze and validate the biological relevance of LotP on Lon protease activity, a new assay was designed. SulA is known to become insoluble in the *E. coli* cytoplasm when it is highly expressed (Ishii et al., 2000). We used a chimerical SulA substrate to measure Lon’s protease activity that does not affect *E. coli* growth. The chimerical substrate was done using SulA carboxyl terminal (11 amino acids) fused to the maltose-binding protein for further purification (MBP.SulA). The results obtained indicated faster degradation of MBP.SulA in the presence of LotP (Figure 8A, lane 3) when compared with the control (Figure 8A, lane 2). Once His.EK.LotP (dimer) was co-expressed with Lon, the MBP.SulA band (Figure 8B, lane 4) at 45 min was similar to the Lon control (Figure 8B, lane 2). These results suggest that the modified protein, His.EK.LotP, had no additional affect over Lon protease activity.

### LotP Affects Photosynthesis Efficiency

The ability of LotP to be highly expressed in citrus plant during ‘*Ca* L. asiaticus’ pathogenicity and its capability to interact with a variety of chaperones led us to assess its potential effects on the citrus plant response.

**FIGURE 9:**
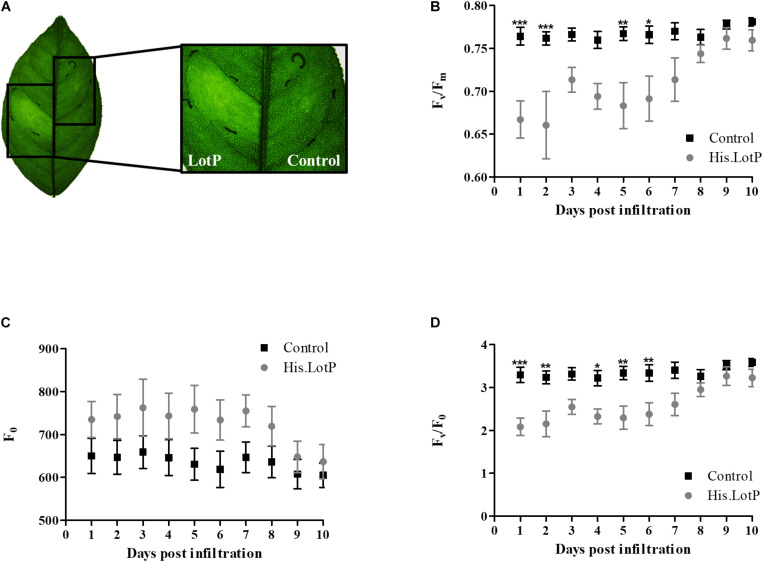
His.LotP causes chlorosis and affects stress levels within citrus. **(A)** Chlorotic effect of His.LotP on citrus leaf in comparison with the vehicle buffer control. *Citrus sinensis* cv. Valencia plants were exposed to purified His.LotP (8 μM) protein, and photosystem II activity was estimated using the chlorophyll fluorescence parameters F_*v*_/F_*m*_ **(B)**, F_0_ **(C)**, and F_*v*_/F_0_ **(D)**. Measurements taken with vehicle control samples are in black squares, while His.LotP measurements are in gray circles. Data are averages ± SEM (*n* = 7). Differences were identified using two-way ANOVA and Tukey’s post-hoc test. Significance is shown as stars: *p < 0.05, **p < 0.01, ***p < 0.001.

Healthy citrus plant leaves were infiltrated with purified His.LotP protein at different concentrations. His.LotP infiltration produced a chlorotic phenotype clearly visible at 3 dpi (days post infiltration) (Figure 9A). To study whether photoinhibition was responsible for chlorosis development, photosynthetic efficiency of the PSII (Fv/Fm) was measured. This index is a sensitive indicator of plant photosynthetic performance and can be used as a direct indication of plant stress and/or photoinhibition. In fact, a decrease in F_*v*_/F_*m*_ has been observed in HLB-infected leaves (Sagaram and Burns, 2009; Cen et al., 2017). Three different concentrations of His.LotP were infiltrated into citrus leaves. Infiltrated His.LotP demonstrated a dose-dependent effect in a range of 4–12 μM (Supplementary Figure 6A). A concentration of 8 μM was selected as the minimal protein concentration required for a reproducible and measurable response. After His.LotP infiltration, F_*v*_/F_*m*_ decreased significantly in the first 2 dpi compared with the values measured in a different lobe of the same leaf that was infiltrated with the vehicle control. A zigzag pattern was displayed over a 10-day period until F_*v*_/F_*m*_ levels reached values comparable with those of the control (Figure 9B). The ground fluorescence (F_0_) remained relatively constant during the assay (Figure 9C); however, the F_*v*_/F_0_ followed a similar zigzag pattern until it reached the levels similar to those of the control at 9 dpi (Figure 9D). These results clearly demonstrated a decreased photosynthetic rate after His.LotP infiltration. In order to evaluate if LotP oligomerization could be responsible of such effects, several citrus leaves were infiltrated with the mutant protein His.EK.LotP.FLAG. No significant changes in the F_*v*_/F_*m*_ parameter were found using this modified protein (Supplementary Figure 6B). This suggested that the biological activity of LotP depends on the ability to form high molecular complexes. Altogether, analysis of LotP infiltrations suggests that the protein may interfere with the proper chloroplast function in citrus leaves, provoking a chlorotic phenotype—two main features observed in HLB-infected plants (Bové, 2006).

### A Quantitative TMT11 MS/MS Analysis Revealed the Citrus Leaf Response to LotP Infiltration

The symptoms displayed after His.LotP infiltration prompted us to evaluate its overall effect on the plant proteomic response. To maximize the possibilities to identify changes in the proteomic profile, samples collected during the first 3 days of the response were evaluated. The infiltration assay was repeated using a solution containing 8 μM of His.LotP in identical conditions as described before. The F_*v*_/F_*m*_ was measured every day, and leaf samples were collected during the first 3 dpi. Collected samples were processed and analyzed with TMT11 MS/MS. The FDR was calculated using the Percolator algorithm in the Proteome Discoverer workflow based on the search results against a decoy database and was set at 1% FDR. Duplicates were grouped, and statistical analyses evaluated as the quantitative ratio between each control and treatment per day. Furthermore, proteins identified, which contained at least two peptides with a *p*-value of <0.05 and log2 fold ratio greater or less than 0.5, were used for final analyses. Overall, a total of 14,958 unique proteins were identified.

**FIGURE 10:**
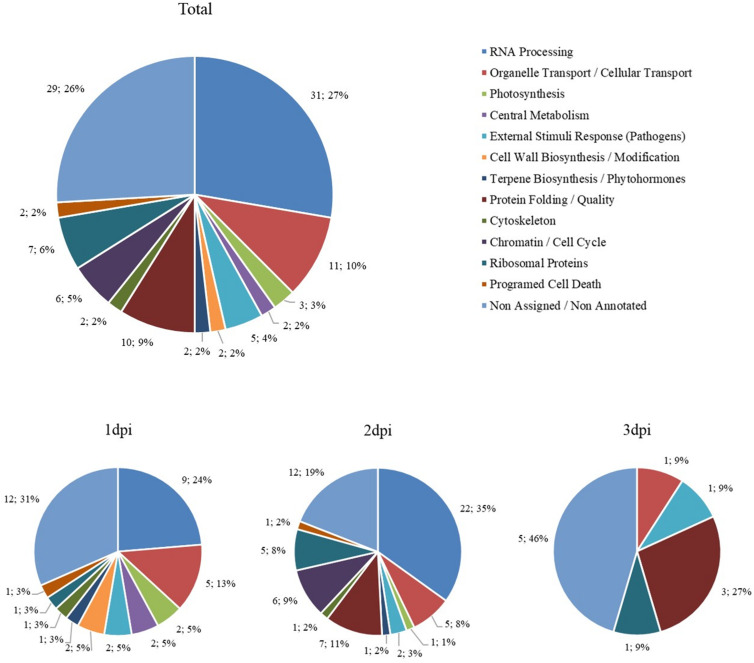
Biological groups of more abundant proteins. The number of differentially more abundant proteins when comparing His.LotP with the control, represented by their biological group. Data are represented as the total amount and percentage of proteins more abundant over 3 days, as well as 1, 2, and 3 dpi.

**FIGURE 11:**
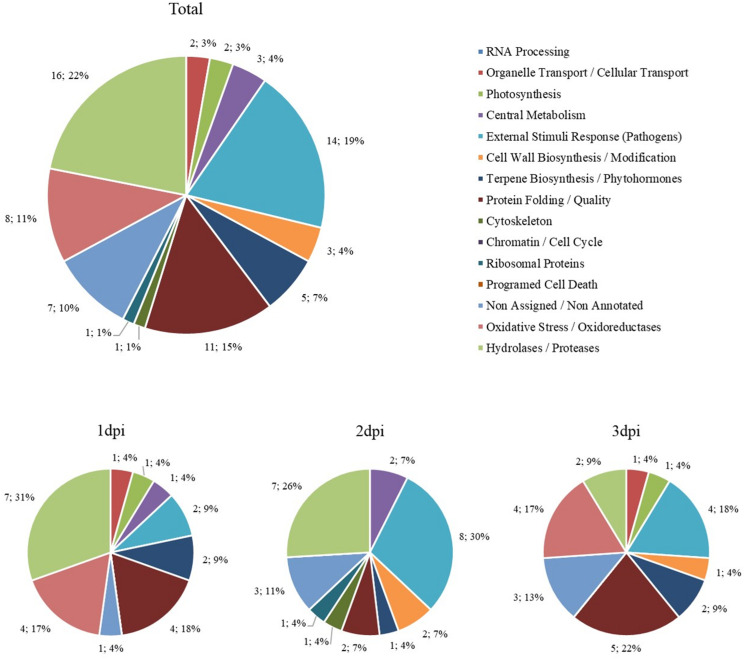
Biological groups of less abundant proteins. The number of differentially less abundant proteins when comparing His.LotP with the control, represented by their biological group. Data are represented as the total amount and percentage of protein less over 3 days, as well as 1, 2, and 3 dpi.

A total of 220 proteins were found to be differently abundant in the period analyzed (first 3 dpi) (Supplementary Table 2). The subsets of proteins with significant differences compared with the control were grouped according to their biological roles and separated by more and less abundant proteins (Figures 10, 11). To better describe the plant response after His.LotP infiltration, the proteins identified were divided into three groups, namely, immediate response (1 dpi), intermediate response (2 dpi), and delayed response (3 dpi) (Table 2). These names do not describe a canonical plant response; they were merely used to group the proteins identified in the window of time examined.

**TABLE 2:** Plant protein responses to His.LotP over time.

	1 dpi		2 dpi		3 dpi	
Plant response/proteins identified	More abundant	Less abundant	More abundant	Less abundant	More abundant	Less abundant
Immediate response	49	23				
Intermediate response			51	27		
Slow plant response					11	23

*Breakdown of proteins found to be differentially abundant based on an immediate (1 dpi), intermediate (2 dpi), or slow plant response (3 dpi) as well as their changes in abundance.*

A subset of 49 proteins significantly increased their abundance at 1 dpi. This group is characterized by the abundance of RNA processing proteins involved in cleavage capping and mRNA polyadenylation. The proteins included in this group were CPSF30, HMG-B (high motility group B proteins), LE ribonuclease, CPFS6, polyadenylate-binding protein 11 (PAIP), and UBP1, suggesting that the plant immediate response is directed to enhance the expression and stabilize critical mRNAs. Interestingly, we also found CPB20/80. These cap binding proteins are involved in the biogenesis of miRNA controlling several plant processes (Kim et al., 2008). With less intensity, but consistently induced, the plant specific PROS regulator was also highly represented. PROS activates SKD1 ATPse activity of the ESCRT III system, controlling the endosomal vesicle trafficking in plants. Two chloroplast proteins were also highly induced after LotP infiltration. Those proteins were part of the Calvin cycle and the photosystem I, supporting the impact of LotP on photosynthesis, as described above. Curiously, APX and Cu/ZnSOD, two important members of the reactive oxygen species scavenging system, were repressed together with a group of glycosylases, peptidases, and oxidases.

The response observed at 2 dpi was less intense in terms of peptide abundance but consists of a larger variety of proteins when compared with the 1 dpi response. Likewise, the RNA processing proteins were dominant. In this group, we also detected a significant increase in the number of transcription factors that respond to biotic and abiotic stresses (Alfin, C2H2-ZF, NTL9, and TCP). Interestingly, GeBP and *Cs*WRKY21 regulators, highly abundant 1 dpi, reached statistical significance at 2 dpi. There was, as well, a significant abundance of proteins involved in protein quality control. These include the lectin chaperone CRT, the HPS60 co-chaperone HSP20, and HSP90. A subset of proteins involved in organelle reconstruction and cell transport were also identified together as well as proteins involved in cell cycle control and autophagy. Hydrolases involved in carbohydrates metabolism, oxidoreductases, and a subgroup of poorly characterized proteins described as potential defensins were negatively affected by the presence of LotP.

Only 11 proteins were observed to be differentially abundant in leaves collected at 3 dpi. This observation relates to a recovery of the PSII activity as previously described. It is important to indicate that three of them were chaperones, while the transcription factors GeBP and *Cs*WRKY21 were still highly expressed. Consistently with the less abundant proteins described in the first two groups, defensins, dehydrogenases and oxidoreductases were negatively affected.

The chimerical His.EK.LotP.FLAG, a protein mainly forming dimers, was used in a parallel assay. As it was expected, the pattern of proteins affected was similar, but the intensity of the response for each protein affected was less intense judging by the relative abundance of the proteins detected. These results reinforce the idea that biological activity of LotP depends on the ability to form high-molecular-weight multimers.

## Discussion

The aim of our study was to assess conformational changes of LotP and to evaluate its potential contribution to ‘*Ca.* L. asiaticus’ pathogenesis. LotP is able to interact with bacterial proteins, like GroEL (Loto et al., 2017), to modify their activity. The evidence collected here indicates that LotP oligomerization is necessary to exert its biological role and interactions. We surmise that both arginine 104 and the N-terminal of LotP are important for efficient dimerization. Deletion of the N-terminal and the R104A mutation (Figures 1C, 4A) generated a switch from dimers to monomers. The residue R104 itself is localized within the globular N-terminal subdomain of the predicted overall structure of LotP (Figure 1D). R104 is facing the linker region between the N- and C-terminal domains. These results suggest that the mutation R104A changes the flexibility between each domain, increasing the resistance to proteolytic degradation (Fontana et al., 2004) and the inability to form dimers.

We identified a new member of the LotP interactome, the Lon protease. It allowed us to evaluate mechanisms of protein–protein contact as well as the biological consequences caused by LotP interaction. Our results demonstrated that both proteins’ N-terminal domains are critical for interaction since a ΔNLon protein decreased its interaction with LotP by 50% (Figure 6A). The importance of the N-terminal region in the formation of Lon’s active proteolytic complex has been previously described (Kereïche et al., 2016). Interestingly, the LotP–Lon protease complex showed increased proteolytic activity toward Lon’s natural targets (SulA) when expressed in *E. coli* (Figure 8). The *E. coli* Lon protease can form hexamers and dodecamers when active in the cytoplasm. The hexamer is shown to be more active, degrading proteins of high molecular weight. In this case, the proteolytic chambers are more exposed to the solvent, thereby increasing its hydrolytic activity. In the case of the dodecamer, the protease chambers of each hexamer are facing each other, decreasing the overall enzymatic activity. It is proposed that this hexamer–dodecamer equilibrium is critical to regulate its activity *in vivo* depending on the growth conditions and the cellular needs (Vieux et al., 2013). Our results suggest that LotP may shift from dimer to multimers, achieving different equilibriums as it was described for the *E. coli* Lon protease, a mechanism that clearly affects the catalytic ability of the Lon protease.

Proteolytic activity is essential to protect cells from unfolded, misfolded, or damaged proteins. Tight regulation of protease activity, as well as an increase or decrease in the synthesis of a protein, can change due to environmental or developmental cues (Kuroda, 2006). LotP can regulate the Lon protease and perhaps the activity of other proteostasis network constituents, such as GroEL, ClpX, and DnaJ, which were all found in co-immunoprecipitation assays (Loto et al., 2017). In parallel, the Lon protease controls the intracellular pool of transcription factors affecting the virulence of several bacterial species (Choi and Groisman, 2020). As *lotP* was initially shown to increase its expression when ‘*Ca.* L. asiaticus’ is transferred from insects to plants (Yan et al., 2013), this type of regulation would allow the bacterium to quickly change its pool of regulatory proteins when moving from the insect vector to the citrus phloem. GroEL is a known LotP target. In mycoplasma, GroEL is displayed within the surface of the bacteria (Hagemann et al., 2017), playing a critical role in endosymbiosis with *Buchnera aphidicola* (Chong et al., 2019). Similarly, ‘*Ca.* L. asiaticus’ is an endosymbiont, and the individual cells thriving inside the citrus phloem displayed a mycoplasma-like morphology (Achor et al., 2020).

With the urgency to understand ‘*Ca.* L. asiaticus’ pathogenicity, we evaluated potential effects of LotP directly on citrus leaves based on several assumptions. We consider that LotP may interact with the plant chaperones, a family of proteins highly conserved across all biological kingdoms. Photosynthesis inhibition associated with an enhancement of the biosynthesis of proteins directly involved in stress response is the consensus model of plant response to ‘*Ca.* L. asiaticus’ infection (Nwugo et al., 2013). LotP leaf infiltration was able to trigger a similar chlorotic phenotype close to the infiltration point affecting plant photosynthesis.

The proteomics data presented in Figures 10, 11 revealed the overall changes in biological protein group abundance over time and between more and less proteins and their respective biological groups. For more abundant proteins (Figure 10), we see that many biological groups (RNA Processing, Ribosomal Proteins, Organelle Transport/Cellular Transport, Chromatin/Cell Cycle, and Protein Folding) are overrepresented at 2 dpi. Meanwhile, for less abundant proteins (Figure 11), there is more continuity over time. However, there is a larger representation of the Photosynthesis group at 1 and 2 dpi and the largest External Stimulus Response (Pathogens) present at 2 dpi.

Interestingly, the proteomic assay demonstrated that RNA binding and RNA editing proteins were predominant in the subset of highly abundant proteins expressed in response to the assault of LotP. This kind of proteins directed to protect and edit RNA molecules are critical to re-establish tissue functionality once it is affected by pathogens or necrotic processes (Woloshen et al., 2011). This response is typically described in plants exposed to diverse types of environmental stress; however, many of their aspects remain poorly characterized. The biogenesis and function of plant organelles are also regulated, like editing and splicing, at a post-transcriptional level (del Campo, 2009; Stern et al., 2010). A similar subset of these proteins was identified in 8 year-old Valencia citrus plants infected with citrus greening (Yao et al., 2020). In parallel to the increased abundance of the RNA stabilizing proteins, we also observed a significant increase of transcriptional regulators responding to biotic and abiotic stress like WRKY21 and GeBP. The WRKY family of transcriptional regulators is involved in a variety of defense responses. Members of this family coordinate PAMP responses, activating phosphorylation cascades and stimulating defense de-repression in plants (Peng et al., 2008; Chen et al., 2019). They also mediate the induction of callose synthase genes as well as jasmonate and salicylic acid biosynthesis in plants reacting against *Pseudomonas syringae* infection (Peng et al., 2008; Park and Ronald, 2012; Chen et al., 2019). Citrus plants encode more than 50 WRKY factors (Vives-Peris et al., 2018) in their genomes. The *Cs*WRKY21 was identified in our proteomic data and shows homology to *At*WRKY50. In the model plant *Arabidopsis thaliana*, this transcription factor modulates the expression of the PR1 (pathogens response 1 gene) (Hussain et al., 2018). Interestingly, our data showed that Prb1 (pathogens resistance b1) was included in the top 200 proteins with statistically significantly higher abundance and showed a clear induction trend of 2 dpi (Table 2). Prb1 production is known in pathogenic relationships such as *Chalara elegans* in tobacco (Tahiri-Alaoui et al., 1990). This transcription factor was also one of the two members of the WRKY family overexpressed in *C. sinensis* infected with ‘*Ca.* L. asiaticus’ and tristeza virus (Fu et al., 2017). The NAC transcription factor identified showed homology to *At*NTL9 where it modulates the expression of PR1 as well (Guo et al., 2017). GeBP regulates a set of genes of the CPR5 pathway particularly involved in pathogen response (Perazza et al., 2011). Members of the Alfin family of transcription factors are triggered by the presence of pathogens as it was described in *Brassica oleracea* in response to *Pectobacterium carotovorum* (Kayum et al., 2016).

Early studies of the ‘*Ca.* L. asiaticus’ genome described a handful of potential pathogenicity determinants or PAMP triggering proteins (Fagen et al., 2014). However, these studies are unable to fully explain the phenotype and the plant responses elsewhere discussed. Since ‘*Ca.* L. asiaticus’ is unculturable, it is impossible to develop knockout mutants for *in vivo* studies. This impedes a direct association of specific genes with the phenotypes observed to claim a full identification of pathogenicity determinants for this disease. Thus, qRT-PCR and mRNA sequencing assays were the techniques of choice to evaluate this pathosystem (Kim et al., 2009). Those studies fell short to predict proteins’ biological roles during ‘*Ca.* L. asiaticus’ infection based on arrays of data.

While some proteins did not reach statistical significance in our assay, we find it important to mention that several showed a solid trend consistent with upregulation. For example, some components of the photosystem I were more abundant compared with the control, while components of the PSII were negatively affected. This observation agrees with the phenotype observed once LotP is infiltrated in the leaves. The ribulose 1–5 biphosphate and five miraculin-like proteins are highly abundant as well (Supplementary Table 2). The biological role of miraculins is related with their ability to inhibit proteolytic enzymes. It is interesting to note that LotP may enhance the citrus tree proteolytic activity like with the aforementioned bacterial Lon protease. *C. sinensis* encodes several Lon proteases, and at least one of them is critical in organelle development and maintenance (Figaj et al., 2019). This enhancement of the serine protease activity may help to explain the results recently published by Franco et al. (2020). These authors described a specific increase of serine protease activity in the leaf extracts of citrus greening-infected plants but no significant changes in the expression of genes encoding citrus proteases (Franco et al., 2020). Overall, the protein expression pattern triggered only by LotP is, in part, consistent with those previously described in ‘*Ca.* L. asiaticus’-infected trees (Fan et al., 2011).

Low abundance proteins were also identified in our assay, but it is more difficult to evaluate and discuss the potential role of this proteins with the experimental design used. Consistently with the chlorotic response detected after infiltration, proteins related with photosynthesis showed lower abundance at 1 dpi. The negative effect on hydrolases and oxidoreductases could be associated with the low metabolic activity of the affected tissue. Interestingly, a group of putative defensins, iron-dependent catalases, and iron-dependent oxidoreductases showed a consistent low abundance at 2 and 3 dpi. These results suggest that the tissue still retains low levels of activity (3 dpi) for the proteins typically synthesized to resume the aftereffects of the hypersensitive plant response. Further assays are necessary to evaluate these effects with more details. Transcriptomics assays will be necessary to elucidate if these low abundance proteins are negatively regulated at the transcriptional level.

In summary, the LotP encoding gene is highly expressed when ‘*Ca.* L. asiaticus’ infects the citrus plants (Yan et al., 2013). In this work, it was proven that the infiltration of the purified protein was able to affect the integrity of citrus plant tissue. While the plant tissues recovered from the assault, we revealed a large abundance of plant proteins associated with the stabilization and processing of mRNA transcripts, a subset of transcription factors modulating the expression of genes, and pathways associated with innate plant defense to be highly expressed. The mechanisms by which LotP exerts its activity were linked to the ability to form large complexes mediating protein–protein interactions. The characteristics of substrate binding module of LotP suggest that this protein may be able to interact with several plant proteins, mainly with chaperons displaying proteolytic activities. The precise identification and characterization of those targets are the subject of future investigation.

## Data Availability Statement

The datasets presented in this study can be found in online repositories. The names of the repository/repositories and accession number(s) can be found below: http://www.proteomexchange.org/, PXD020912.

## Author Contributions

CG and GL acquired the funding as well as contributed resource provisions. CG, MMe, and KP-P conceptualized the work. MMe, KP-P, AC, and LG conducted the research. KP-P performed the data curation and formal analysis. CG, GL, and MMa provided the mentorship and advice for research. MMe, KP-P, and CG did curation of the published work. All the authors contributed to the article and approved the submitted version.

## Conflict of Interest

The authors declare that the research was conducted in the absence of any commercial or financial relationships that could be construed as a potential conflict of interest.

## Publisher’s Note

All claims expressed in this article are solely those of the authors and do not necessarily represent those of their affiliated organizations, or those of the publisher, the editors and the reviewers. Any product that may be evaluated in this article, or claim that may be made by its manufacturer, is not guaranteed or endorsed by the publisher.

## Footnotes

1
https://blast.ncbi.nlm.nih.gov/Blast.cgi

2
https://embnet.vital-it.ch/software/BOX_form.html

## Supplementary Material

Supplementary Figure 1 Conserved amino acids across LotP domains. Alignment of ‘*Ca*. L. asiaticus’ LotP sequence (#1) with homologs encoded in phylogenetically close organisms (#2–10) and phylogenetically distant organisms (#11–14). Alignment with homologs encoded in the order Rhizobiales – (#2–10) and in the order Xanthomonadales (#11–14). Mutated residues are depicted with an asterisk (^∗^). Bacterial species: (1) ‘*Ca*. L. asiaticus,’ (2) ‘*Ca*. L. solanacearum,’ (3) ‘*Ca.* L. africanus,’ (4) ‘*Ca*. L. americanus,’ (5) *L. crescens*, (6) *Rhizobiales bacterium*, (7) *Agrobacterium tumefaciens*, (8) *Rhizobium grahamii*, (9) *Mycoplana dimorpha*, (10) *Rhizobium leguminosarum*, (11) *Arenimonas composti*, (12) *Pseudoxanthomonas mexicana*, (13) *Stenotrophomonas rhizophila*, (14) *Xanthomonas campestris*.

Supplementary Figure 2 Control of LotP oligomers. Representative chromatogram obtained of His.LotP and LotP.His analyzed by size-exclusion chromatography using a Superose 6 column. The purified proteins were separate according to its native molecular size. Elutions were monitored continuously at 280 nm and the graphic shows the signal at the different elution volumes.

Supplementary Figure 3 Mutation R104A causes structural changes to LotP. SDS-PAGE of samples of purified His.LotP, His.LotP^R104A^, His.EK.LotP, ΔNLotP.His, and His.EK.LotP^R104A^ proteins analyzed by a limited proteolysis assay. Purified proteins were incubated with proteinase K and aliquots were taken at sequential time points (0, 5, 15, 45, 90, and 240 min) and the degradation was halted with the addition of PMSF. The assay was done in duplicate while a representative gel is shown in the figure. 3.5 μg of protein were used per time point.

Supplementary Figure 4 LotP and N-terminal domain of Lon proteases Alignment. Alignment of ‘*Ca*. L. asiaticus’ LotP sequence (#1) with N-terminal domains of Lon proteases (#2–4). Bacterial species per line is as follows: (1) ‘*Ca*. L. asiaticus’ LotP, (2) ‘*Ca*. L. asiaticus’ Lon, (3) *Escherichia coli* Lon, and (4) *Bacillus subtilis* Lon.

Supplementary Figure 5 Control of protein expression in growth curves by Western blot. **(A,B)** Western blot using anti-LotP antibodies with samples taken from cultures of wt and Δ*lon E. coli* strains overexpressing LotP **(A)**, LotP^R104A^ **(B)**, or those transformed with the empty pBAD24 vector as a control. **(C)** Western blot with anti-His antibodies and SDS-PAGE stained with Coomassie blue of samples taken from the growth curves. Figures A-C correspond to samples from growth curves in exponential growth phase from Figures 7A–C, respectively. The assay was done in triplicate while a representative western blot is shown in the figure.

Supplementary Figure 6 Concentration and conformation effects of LotP on plant stress. **(A)** *Citrus sinensis* cv. Valencia plants were exposed varying concentration of His.LotP: 4 μM (medium gray), 8 μM (black), and 12 μM (light gray). **(B)** *Citrus sinensis* cv. Valencia plants were exposed His.LotP (circle) and His.EK.LotP.FLAG (triangle) at 8 μM. Photosystem II activity was estimated using the chlorophyll fluorescence parameter F_*v*_/F_*m*_. Data are averages ± SEM (*n* = 7). Differences were identified using two-way ANOVA and Tukey’s *post hoc* test.
