# Supplementary figures and images for: ‘Candidatus Liberibacter asiaticus’ Multimeric LotP Mediates Citrus sinensis Defense Response Activation
Source: Front Microbiol. 2021 Aug 4;12:661547. doi: 10.3389/fmicb.2021.661547 (PMC8371691; doi:10.3389/fmicb.2021.661547)

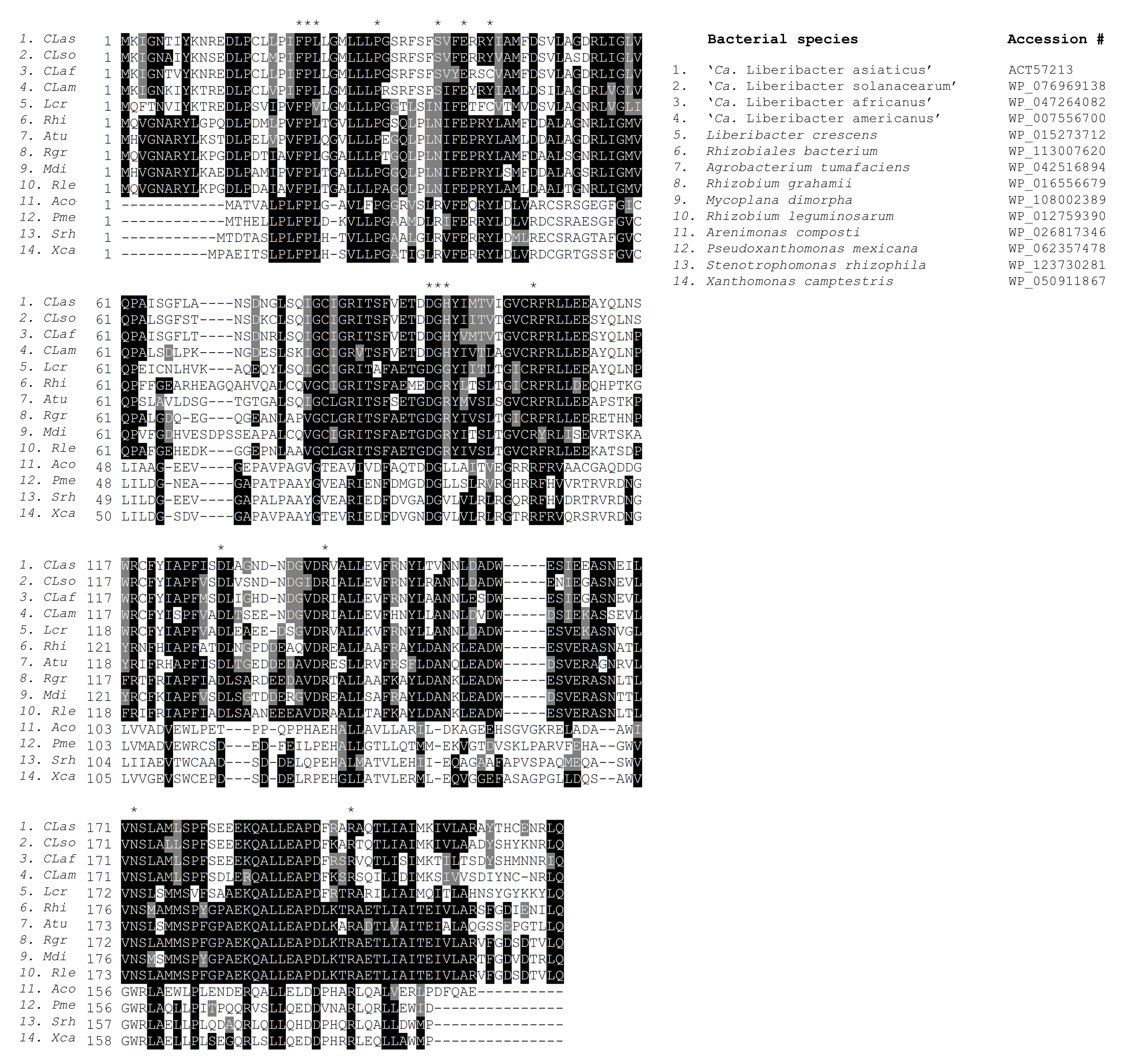

Supplement: Supplementary Figure 1 — Conserved amino acids across LotP domains. Alignment of ‘Ca. L. asiaticus’ LotP sequence (#1) with homologs encoded in phylogenetically close organisms (#2–10) and phylogenetically distant organisms (#11–14). Alignment with homologs encoded in the order Rhizobiales – (#2–10) and in the order Xanthomonadales (#11–14). Mutated residues are depicted with an asterisk (∗). Bacterial species: (1) ‘Ca. L. asiaticus,’ (2) ‘Ca. L. solanacearum,’ (3) ‘Ca. L. africanus,’ (4) ‘Ca. L. americanus,’ (5) L. crescens, (6) Rhizobiales bacterium, (7) Agrobacterium tumefaciens, (8) Rhizobium grahamii, (9) Mycoplana dimorpha, (10) Rhizobium leguminosarum, (11) Arenimonas composti, (12) Pseudoxanthomonas mexicana, (13) Stenotrophomonas rhizophila, (14) Xanthomonas campestris. [file Image_1.tif]

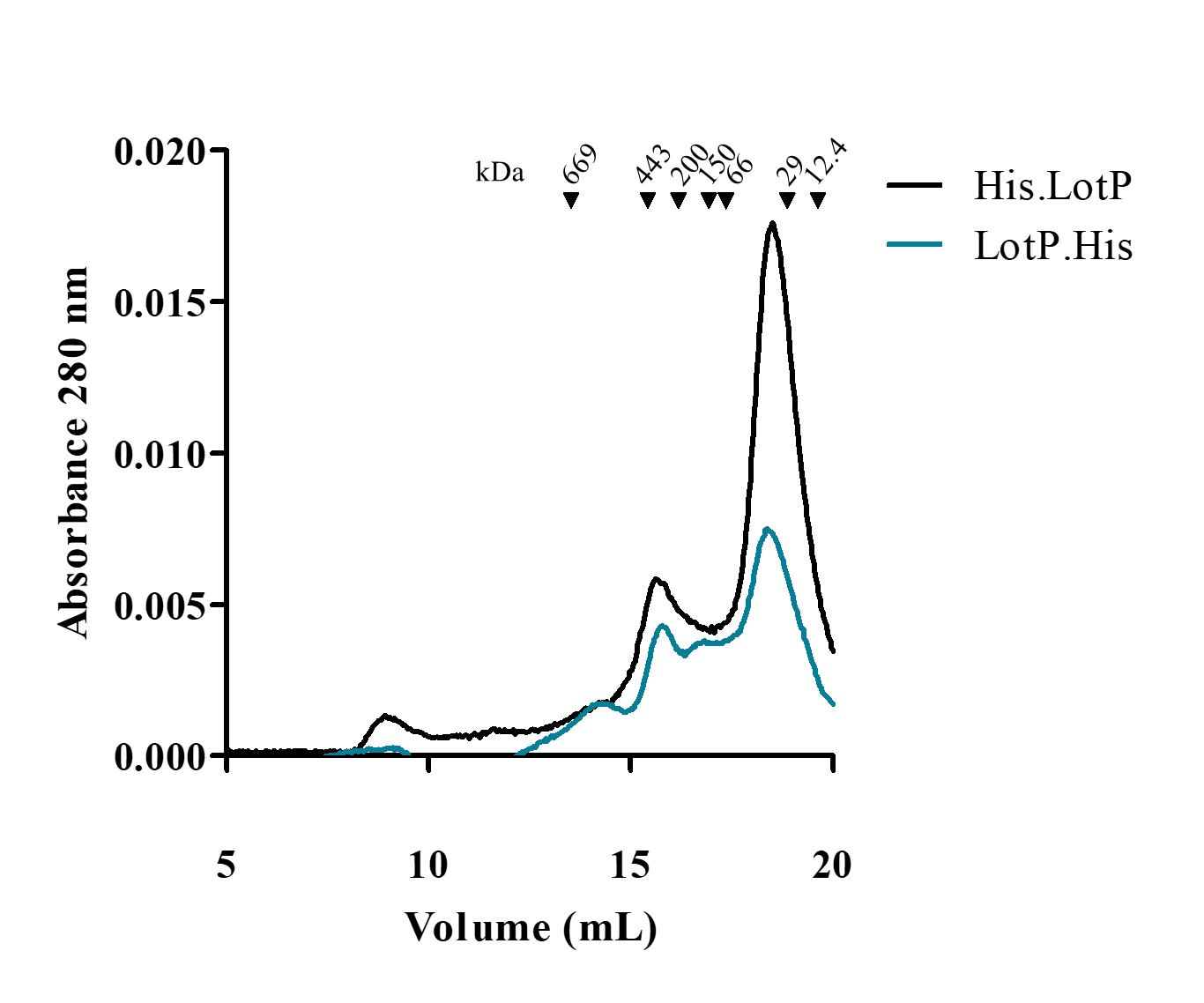

Supplement: Supplementary Figure 2 — Control of LotP oligomers. Representative chromatogram obtained of His.LotP and LotP.His analyzed by size-exclusion chromatography using a Superose 6 column. The purified proteins were separate according to its native molecular size. Elutions were monitored continuously at 280 nm and the graphic shows the signal at the different elution volumes. [file Image_2.tif]

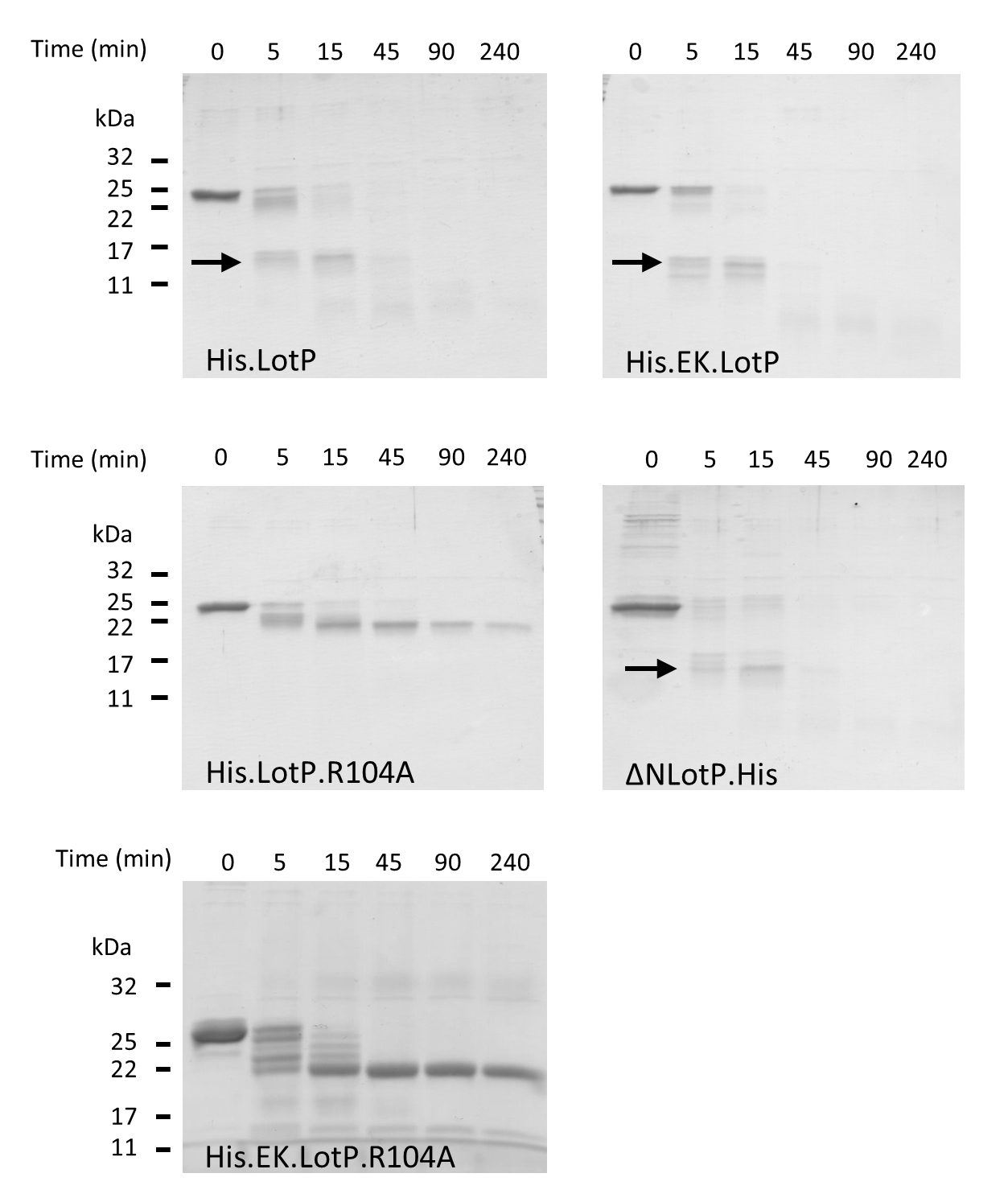

Supplement: Supplementary Figure 3 — Mutation R104A causes structural changes to LotP. SDS-PAGE of samples of purified His.LotP, His.LotPR104A, His.EK.LotP, ΔNLotP.His, and His.EK.LotPR104A proteins analyzed by a limited proteolysis assay. Purified proteins were incubated with proteinase K and aliquots were taken at sequential time points (0, 5, 15, 45, 90, and 240 min) and the degradation was halted with the addition of PMSF. The assay was done in duplicate while a representative gel is shown in the figure. 3.5 μg of protein were used per time point. [file Image_3.tif]

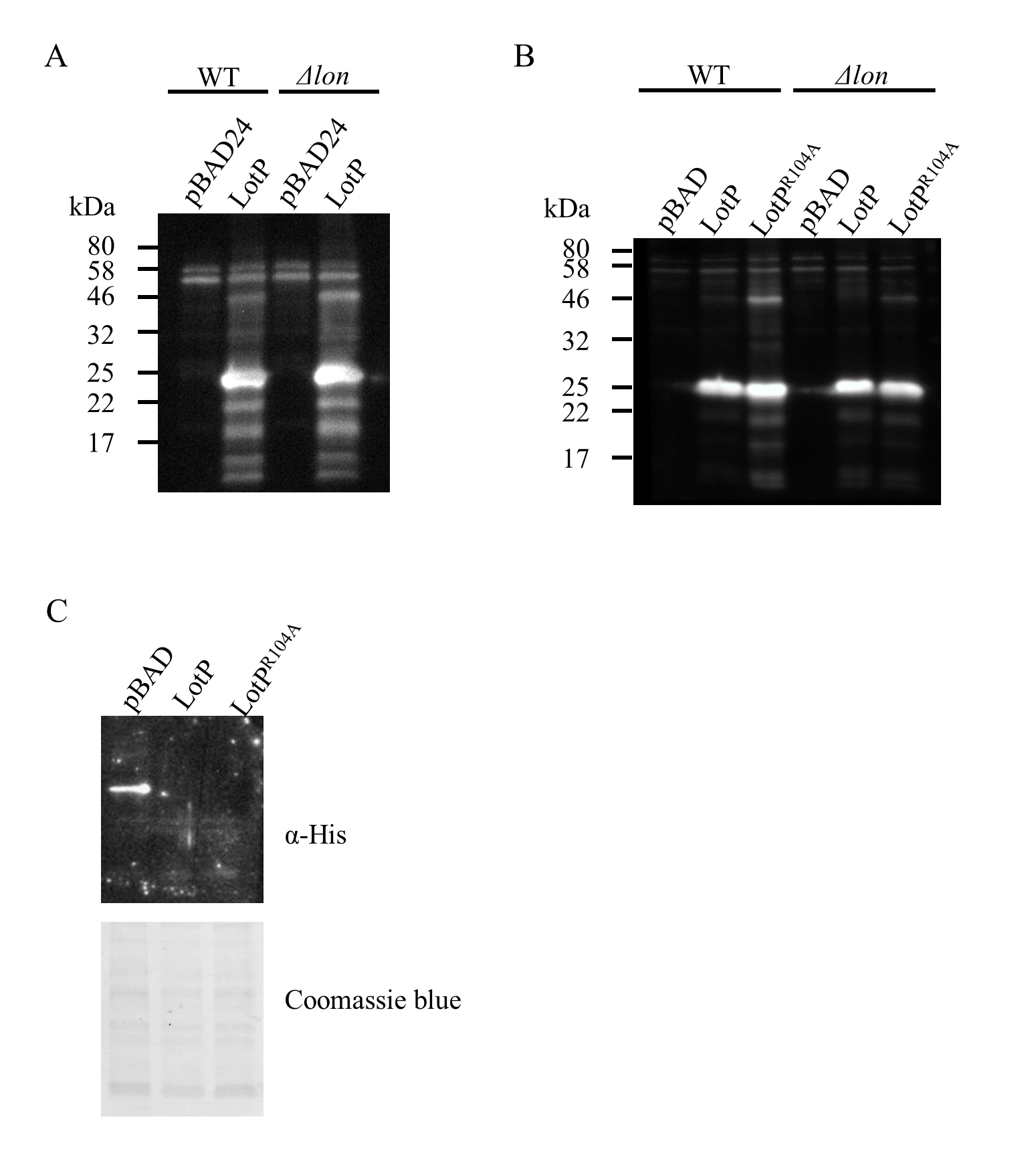

Supplement: Supplementary Figure 4 — LotP and N-terminal domain of Lon proteases Alignment. Alignment of ‘Ca. L. asiaticus’ LotP sequence (#1) with N-terminal domains of Lon proteases (#2–4). Bacterial species per line is as follows: (1) ‘Ca. L. asiaticus’ LotP, (2) ‘Ca. L. asiaticus’ Lon, (3) Escherichia coli Lon, and (4) Bacillus subtilis Lon. [file Image_4.tif]

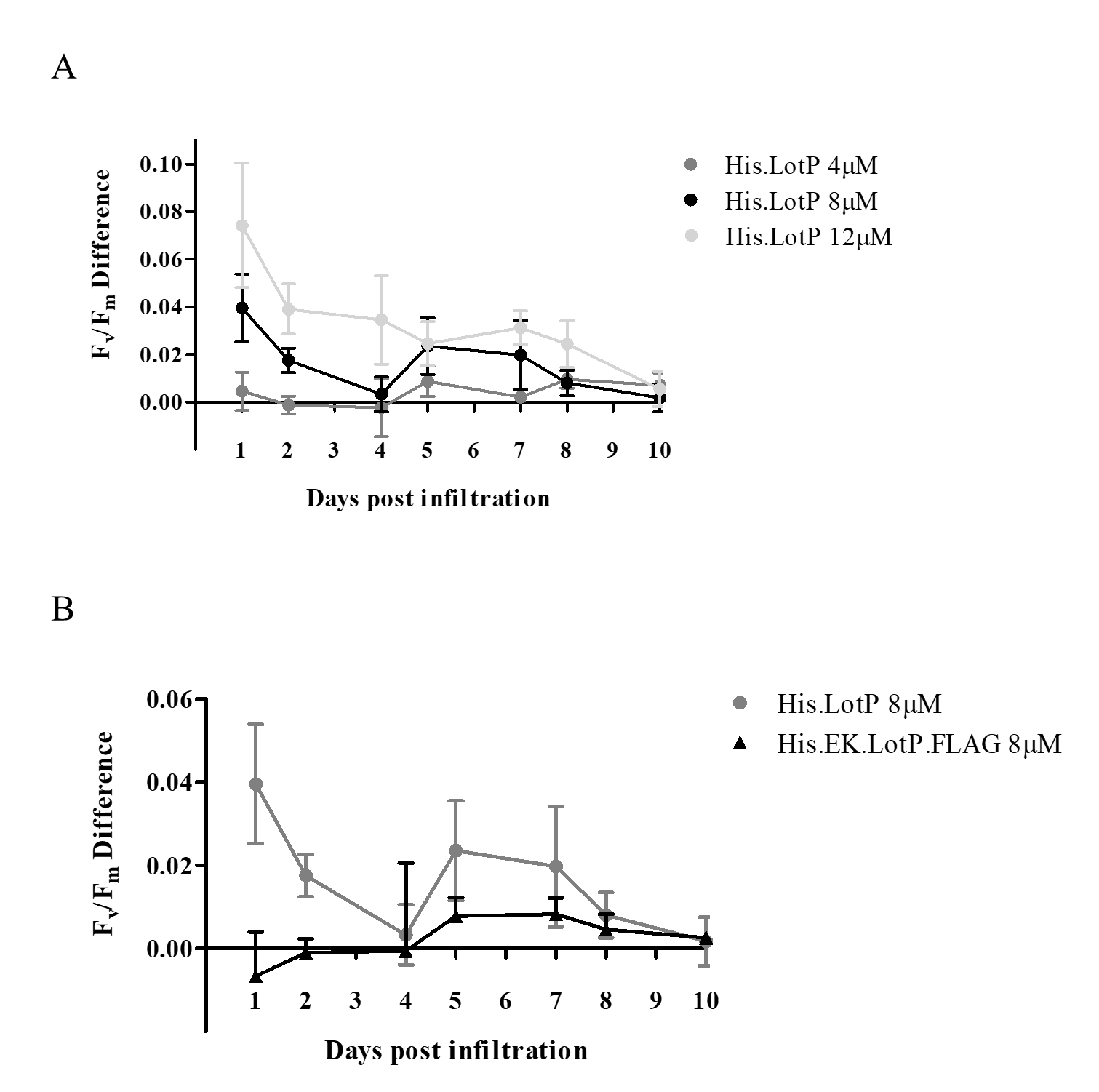

Supplement: Supplementary Figure 6 — Concentration and conformation effects of LotP on plant stress. (A) Citrus sinensis cv. Valencia plants were exposed varying concentration of His.LotP: 4 μM (medium gray), 8 μM (black), and 12 μM (light gray). (B) Citrus sinensis cv. Valencia plants were exposed His.LotP (circle) and His.EK.LotP.FLAG (triangle) at 8 μM. Photosystem II activity was estimated using the chlorophyll fluorescence parameter Fv/Fm. Data are averages ± SEM (n = 7). Differences were identified using two-way ANOVA and Tukey’s post hoc test. [file Image_6.tif]
