# Supplementary material for: ‘Candidatus Liberibacter asiaticus’ Multimeric LotP Mediates Citrus sinensis Defense Response Activation
Source: Front Microbiol. 2021 Aug 4;12:661547. doi: 10.3389/fmicb.2021.661547 (PMC8371691; doi:10.3389/fmicb.2021.661547)
Supplement: Supplementary file 7 [file Table_1.DOCX]

| **Primers by use** |  |  |
| --- | --- | --- |
|  | **Primer Name** | **Primer sequence (5' → 3')*** |
| **Mutagenesis** |  |  |
| LotP_R104A | LotP_R104A_Fw | CTGTAATCGGTGTTTGTgcATTCCGATTGCTAG |
|  | LotP_R104A_Rv | CTAGCAATCGGAATGCACAAACACCGATTACAG |
| **LotP N- and C-terminal modifications** | | |
| His.EK.LotP.FLAG | Fw_His.LotP_*Bam*H | ctgggatccgATGAAAATTGGTAATACAATATAC |
|  | Rv_LotP_FLAGStop_*Xho*I | agctcgagctatttatcatcatcatctttataatcTTGCAACCTATTTTCAC |
| His.EK.LotP.FLAG | Fw_His.LotP_*Bam*HI | ctgggatccgATGAAAATTGGTAATACAATATAC |
|  | Rv_LotP_*Xho*I | agctcgagCTATTGCAACCTATTTTCAC |
| LotP.His | Fw_LotP_*Nco*I | ctgccatggccATGAAAATTGGTAATACAATATAC |
|  | Rv_LotP_HisStop_*Xho*I | agctcgagctaatggtgatggtgatgatgaccTTGCAACCTATTTTCAC |
| ΔNLotP.His | Fw_ΔNLotP_*Nco*I | atccatggccGAAGACTTACCGTGTTTATTG |
|  | Rv_LotP_HisStop_*Xho*I | agctcgagctaatggtgatggtgatgatgaccTTGCAACCTATTTTCAC |
| DDDPD.LotP.His | Fw_LotP+DDD_*Nco*I | cgccatggccgatgatgatgatccggatccgATGAAAATTGGTAATACAATATAC |
|  | Rv_LotP_HisStop_*Xho*I | agctcgagctaatggtgatggtgatgatgaccTTGCAACCTATTTTCAC |
| N-terminal(Lon)-LotP.His | Fw_N1LotP | gaatatgtgacctgcgaagatgaaagcaaagatcgcATGAAAATTGGTAATACAATATAC |
|  | Fw_N3LotP_*Nco*I | ctgccatggccaaccagagcgatgaaaaacgCGAATATGTGACCTGCGAAGATG |
|  | Rv_LotP_HisStop_*Xho*I | agctcgagctaatggtgatggtgatgatgaccTTGCAACCTATTTTCAC |
| N-terminal(Lon)-ΔNLotP.His | Fw_N2LotP | cgaatatgtgacctgcgaagatgaaagcaaagatCGTGAAGACTTACCGTG |
|  | Fw_N3LotP_*Nco*I | ctgccatggccaaccagagcgatgaaaaacgCGAATATGTGACCTGCGAAGATG |
|  | Rv_LotP_HisStop_*Xho*I | agctcgagctaatggtgatggtgatgatgaccTTGCAACCTATTTTCAC |
| **Bacterial two-hybrids**^†^ | |  |
| Lon | Fw_Lon_*Nde*I | ggaattccatatgTTGAATCAAAGCGATGAAAAA |
|  | Rv_Lon_*Not*I | tatatgcggccgcATGAGCTACTGATCTGCCATCTT |
| ΔNLon | Fw_ΔNLon_*Nde*I | gcgcatatggGTATAATCTATCCACTTCTGCC |
|  | Rv_Lon_*Not*I | tatatgcggccgcATGAGCTACTGATCTGCCATCTT |
| His.EK.LotP | Fw_His.LotP_*Nde*I | gcgcatatgGCACATCACCACCACCATC |
|  | Rv_LotP_*Not*I | tatatgcggccgcTTGCAACCTATTTTCACAATGA |
| LotP^R104A^ | Fw_LotP_*Nde*I | ggatccatatgATGAAAATTGGTAATACAATATACAAA |
|  | Rv_LotP_*Not*I | tatatgcggccgcTTGCAACCTATTTTCACAATGA |
| ΔNLotP.His | Fw_ΔNLotP_*Nde*I | gcgcatatgGAAGACTTACCGTGTTTATTG |
|  | Rv_LotP_*Not*I | tatatgcggccgcTTGCAACCTATTTTCACAATGA |
| **Cloning into pBAD24** | |  |
| LotP^R104A^ | Fw_LotP_*Kpn*I | cgatgtggtacctATGAAAATTGGTAATACAATA |
|  | Rv_LotP_*Sal*I | ttctacgtcgactATTGCAACCTATTTTC |
| His.EK.LotP | Fw_HisLotP_*Xho*I | agctcgagATACCATGGCACATCACC |
|  | Rv_LotP_*Sal*I | tacgtcgaCTATTGCAACCTATTTTCACAATGAG |
| **Degradation assay** | |  |
| pCDF-1b_FLAG.MBP.C-terminalSulA | Fw_FLAG.MBD_*Nco*I | ctgccatggcagattataaagatgatgatgataaaATGAAAATCGAAGAAGGTAAACTGG |
|  | Rv_MBD.TEV_*Sac*I | aggagctcgctctggaaatacaagttttcAGTCTGCGCGTCTTTCAG |
|  | Rv_SulAdegron_*Sac*I | aggagctcctaatgatacaggttgctatgaattttcaggccgctGCTCTGGAAATACAAGTTTTC |

*Lower letters were added by PCR amplification. Underlined sequences refer to restriction enzyme sites.
